# Structural basis for substrate and inhibitor recognition of human multidrug transporter MRP4

**DOI:** 10.1038/s42003-023-04935-7

**Published:** 2023-05-22

**Authors:** Ying Huang, Chenyang Xue, Liangdong Wang, Ruiqian Bu, Jianqiang Mu, Yong Wang, Zhongmin Liu

**Affiliations:** 1 Department Of Immunology And Microbiology, School of Life Sciences, Southern University of Science and Technology, Shenzhen, 518055 Guangdong China; 2 College of Life Sciences, Zhejiang University, Hangzhou, 310027 China; 3 The Provincial International Science and Technology Cooperation Base on Engineering Biology, International Campus of Zhejiang University, Haining, 314400 China

**Keywords:** Cryoelectron microscopy, Membranes

## Abstract

Human multidrug resistance protein 4 (hMRP4, also known as ABCC4), with a representative topology of the MRP subfamily, translocates various substrates across the membrane and contributes to the development of multidrug resistance. However, the underlying transport mechanism of hMRP4 remains unclear due to a lack of high-resolution structures. Here, we use cryogenic electron microscopy (cryo-EM) to resolve its near-atomic structures in the apo inward-open and the ATP-bound outward-open states. We also capture the PGE1 substrate-bound structure and, importantly, the inhibitor-bound structure of hMRP4 in complex with sulindac, revealing that substrate and inhibitor compete for the same hydrophobic binding pocket although with different binding modes. Moreover, our cryo-EM structures, together with molecular dynamics simulations and biochemical assay, shed light on the structural basis of the substrate transport and inhibition mechanism, with implications for the development of hMRP4-targeted drugs.

## Abstract (web summary)

Cryo-EM structures, MD simulations, and biochemical assays of human multidrug resistance-associated protein 4 in apo inward-open, ATP-bound outward-open, PGE1 substrate-bound, and sulindac inhibitor-bound states provide insights into substrate transport and inhibition mechanism.

## Introduction

ATP binding cassette (ABC) transporters, a superfamily of membrane proteins ubiquitous in all organisms, transport chemically diverse substrates across lipid membranes by ATP hydrolysis^1,2^. In humans, the ABC subfamily C (ABCC) contains 13 members, of which at least 9 are multidrug resistance (MDR)-associated proteins (MRP), called MRP1 to MRP9^3^. MRPs are responsible for the extrusion of both endobiotics and xenobiotics, playing an important role in both the regulation of physiological processes and the development of MDR^3,4^.

**Fig. 1:**
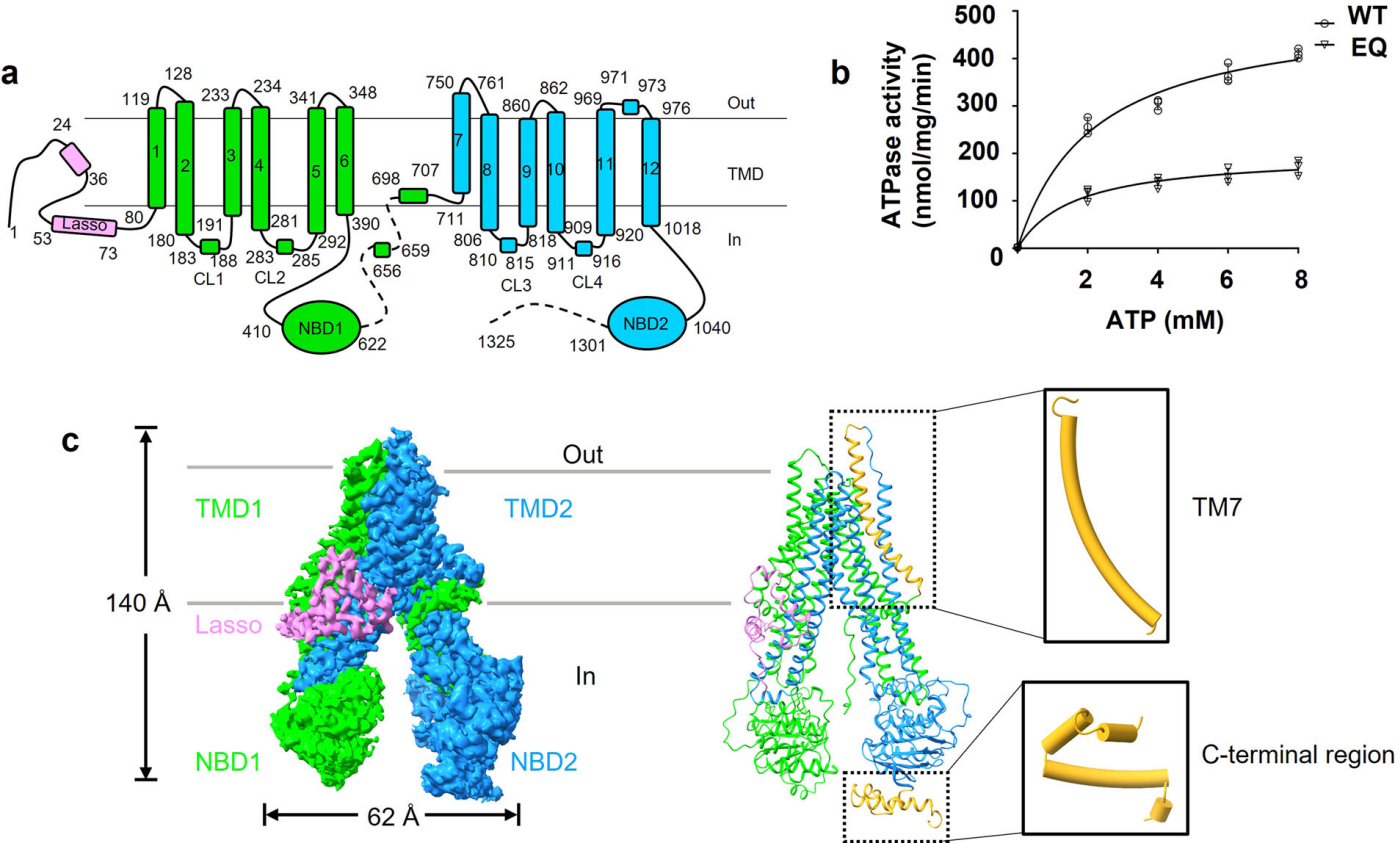
Functional characterization and overall structure of apo hMRP4. **a** Topology diagram of hMRP4. Regions not included in the model are represented by dashed lines. **b** ATPase activity of detergent-solubilized wild-type (WT) and E1202Q hMRP4 (EQ). The data points represent the means of three independent measurements (*n* = 3) and the error bars indicate the mean ± standard deviation. Lines are fitted by nonlinear regression of the Michaelis-Menten equation. Source data are provided in Supplementary Data 3. **c** Cryogenic electron microscopy map of apo-form hMRP4 (left). Cartoon representation of apo-form hMRP4 (right). The lasso domain is colored violet and the two halves of hMRP4 are colored lime and dodger blue for half 1 (transmembrane domain [TMD] 1 and nucleotide-binding domain [NBD]1) and half 2 (TMD2 and NBD2), respectively. Transmembrane helix 7 (TM7) and the C-terminal helices of hMRP4 are colored goldenrod. All structural figures were prepared using ChimeraX 1.4. Contour levels are 0.394 (lasso domain), 0.273 (TMD1 and NBD1) and 0.226 (TMD2 and NBD2).

Human MRP4 (hMRP4), encoded by the *ABCC4* gene, comprises a single polypeptide of 1325 residues (Fig. 1a), which was first identified in human cancer cell lines in 1997^5^. hMRP4 has been found in almost all tissues and cell types^6^ and extrudes a wide variety of antiviral, anticancer, and antibiotic drugs^7^. Moreover, hMRP4 transports various endogenous signaling molecules, including cyclic nucleotides, folic acid, eicosanoids, urate, prostaglandins, leukotrienes, and conjugated steroids^7^. Accordingly, hMRP4 plays an essential role in physiological processes, the dysregulation of which is highly associated with the development of diseases^8^.

According to the Human Gene Mutations Database (www.hgmd.cf.ac.uk), almost 300 missense mutations of hMRP4 are associated with diseases. These pathogenic missense mutations spread over the entire MRP4 region, suggesting that all of its structural domains are functionally important. Mutations of hMRP4 that abolish its transporter function would sensitize LLC-PK1 cells and developing embryos to toxic pesticides^9^. Mutations and dysregulation of hMRP4 are also related to a variety of diseases, including rheumatoid arthritis^10^, leukemia^11^, small cell lung cancer^12^, inflammatory airway diseases^13^, dysfunction of the blood-brain barrier^14^, secretory diarrhea^15^, and cardiovascular disease^16^. Prostaglandin E1 (PGE1) is an endogenous prostaglandin that mediates vasodilation and vasoconstriction^17,18^. Alprostadil, the synthetic form of PGE1, is widely used as a vasodilator in critical congenital cardiac disease to maintain ductal patency and facilitate pulmonary and systemic blood flow^19^. hMRP4 can bind PGE1 with high affinity and translocate it across the membrane^7,20^, thereby influencing the clinical effectiveness of PGE1 and its analogs.

The MRP transport mechanism has been explored using structures of the bovine MRP1 (bMRP1) complex^21–23^; however, hMRP4 notably differs from bMRP1 in composition, architecture, substrate recognition, and function. Indeed, hMRP4 shares <40% sequence identity with bMRP1. Unlike bMRP1, which contains an extra transmembrane domain (TMD) denoted as TMD0, hMRP4 presents a typical ABC transporter structure with two TMDs (TMD1 and TMD2) and two nucleotide-binding domains (NBDs; NBD1 and NBD2). Of note, there is no evidence that bMRP1 has the capacity to transport PGE1^24^. Accordingly, the information deduced from LTC_4_-bound bMRP1 structures is insufficient to understand the mechanism by which MRP4 translocates PGE1.

Given that MRPs play important roles in many essential biological processes by transporting various substrates, inhibiting the transport activity of MRP may facilitate the treatment of MRP-associated diseases. Studies have shown that MRP4 inhibition or deficiency notably diminishes the abundance of the cyclic nucleotide cGMP in mice, and treatment with MRP4 inhibitors enhances cyclic nucleotide-dependent platelet inhibition after PGE1 induction^25^. Moreover, MRP4 inhibitors can inhibit platelet signaling pathway activation and reduce average thrombus size by about 40%^25^. Sulindac, a non-steroidal anti-inflammatory drug (NSAID) that inhibits the prostaglandin pathway by blocking the activity of cyclooxygenases-1 and -2^26^, also reportedly inhibits MRP4^27^, as do many other MRP inhibitors, including salicylate, piroxicam, ibuprofen, naproxen, tolmetin, and etodolac. However, no structures of the sulindac-bound hMRP complex have been reported, and the mechanism underlying sulindac inhibition of hMRP4 is far from clear. Therefore, resolving the structure of hMRP4 will deepen our understanding of its mechanism of action in pathogenic and therapeutic processes. Moreover, considering that hMRP4 shares the most common MRP family topology, understanding the structural basis of hMRP4 function may provide clues to the mechanism of other MRPs.

In this work, we determined the high-resolution structures of hMRP4 in four distinct functional states using single-particle cryogenic electron microscopy (cryo-EM). Integration of the cryo-EM structures, molecular dynamics (MD) simulations, and biochemical results enables us to decipher the mechanisms of substrate translocation and sulindac-mediated inhibition and provides direction for the development of hMRP4-targeted inhibitory drugs.

## Results

### Biochemical characterization and structural determination of hMRP4

To study the biochemical function and structure of hMRP4, we recombined hMRP4 with a C-terminal 3×Flag tag and transiently expressed the construct in HEK293F suspension cells. The hMRP4 protein was extracted from membranes using a mixture of n-dodecyl-β-D-maltoside (DDM) and cholesteryl hemisuccinate (CHS), which was exchanged to digitonin via size exclusion chromatography (SEC). The purified protein appeared homogeneous in the detergent-solubilized micelles (Supplementary Fig. 1a, b). Next, we measured the adenosine triphosphatase (ATPase) activity of wild-type hMRP4 (wt-hMRP4) and its E1202Q mutant in which the catalytic glutamate at position 1202 in NBD2 is replaced by glutamine (hereafter referred to as hMRP4[EQ]). We observed that the EQ mutant did not affect the protein expression (Supplementary Fig. 1c), and the ATPase activity of both proteins increased upon the addition of ATP, but the activity of wt-hMRP4 exceeded that of hMRP4[EQ] (Fig. 1b). The ATPase activity of wt-hMRP4 increased from roughly 400 nmol/mg/min in the absence of PGE1 to ~800 nmol/mg/min in the presence of PGE1 (20 to 100 µM) (Supplementary Fig. 1d). We also observed that the ATPase activity of wt-hMRP4 was higher in the presence of sulindac (Supplementary Fig. 1e), in line with the previous report that high-affinity inhibitors can also accelerate ATP hydrolysis^28^. By contrast, the addition of PGE1 or sulindac did not stimulate the ATP hydrolysis of hMRP4[EQ] (Supplementary Fig. 1d, e). Thus our purified hMRP4 displayed reasonable ATPase activity and was suitable for the following structural studies.

Next, we collected 5411 movies of wt-hMRP4 cryo-EM samples, which were processed using cryoSPARC software, yielding a high-resolution map with an overall resolution of ~3.13 Å (Supplementary Fig. 2). The EM map showed excellent side-chain density (Supplementary Fig. 2e) and enabled us to build an unambiguous model of most regions of hMRP4 (Fig. 1c), with the exception of some flexible loops (residues from 630 to 693 and 1302 to 1325). The overall architecture of hMRP4 presented a typical ABC transporter topology, comprising a lasso domain, two TMDs, and two NBDs, with cytosolic and extracellular loops connecting these domains (Fig. 1a and c). hMRP4 exhibited an inward-open conformation in the absence of ATP (Fig. 1c), as found for other ABC transporters^23,29,30^. The height of the TMDs and NBDs was ~140 Å, and the width between the two NBDs was ~62 Å. TMD1 and TMD2, each consisting of six transmembrane helices (TM), were domain-swapped such that TM4-5 and TM10-11 were packed against each other as in other type IV ABC exporters. The lasso domain, which has been proposed to facilitate proper folding and trafficking of ABCC proteins^23^, was clearly determined near the cytosolic side of MRP4’s transmembrane region, similar to its position in bMRP1 (Supplementary Fig. 3a). However, unlike bMRP1, hMRP4 topologically displays the most common core structure in the MRP subfamily without TMD0 (Fig. 1a and c, Supplementary Fig. 3a).

Of note, hMRP4 TM7 extended to the extracellular region, comprising an elongated helix that is longer than its counterparts in other MRP transporters (Fig. 1c, Supplementary Fig. 3b). In addition, the C-terminus of hMRP4 presented a short helix structure that has not been observed in other MRP members (Fig. 1c, Supplementary Fig. 3b).

### The inward-open structure of substrate-bound hMRP4

**Fig. 2:**
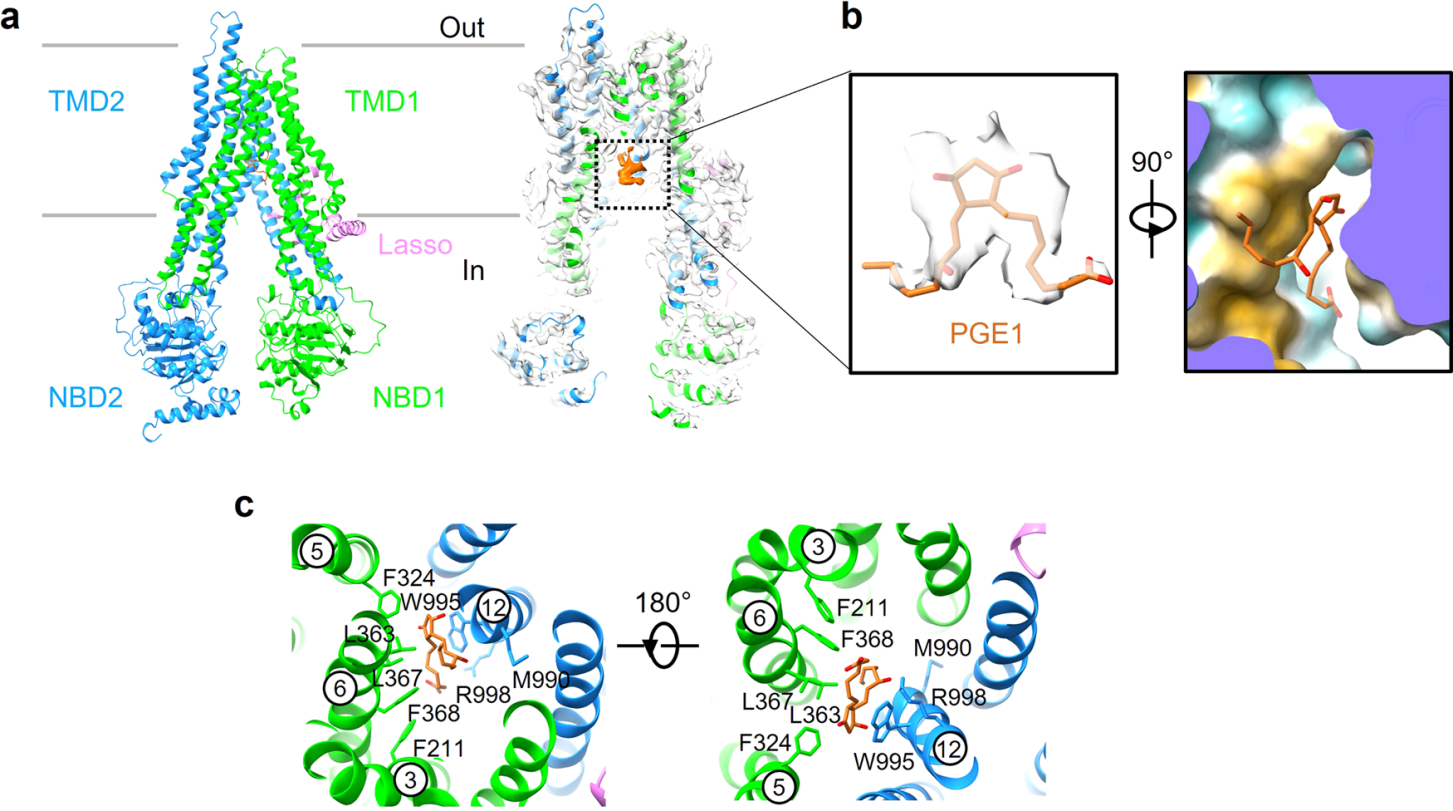
Structural features of prostaglandin E1-bound hMRP4. **a** Cartoon representation of the atomic model of the prostaglandin E1 (PGE1)-bound hMRP4 (left). Cryogenic electron microscopy map of PGE1-bound hMRP4 (right). **b** Electron microscopy density of PGE1 in the same orientation as (A) (left). PGE1 is buried in a pocket formed by the hydrophobic residues of hMRP4 (right). Contour level is 0.308. **c** Coordination of PGE1 by hMRP4. Residues related to substrate binding are shown as sticks. Transmembrane helices (TMs) 3, 5, 6, and 12, which interact with PGE1, are labeled.

Next, to study the mechanism of hMRP4 substrate transport, we incubated hMRP4 with various substrates, including dehydroepiandrosterone (DHEA) and PGE1, to reconstitute a substrate-bound hMRP4 complex. We succeeded in obtaining a substrate-bound hMRP4 complex by incubating apo MRP4 with 100 µM PGE1 on ice for 1 h (Fig. 2). The cryo-EM reconstruction of the PGE1-bound hMRP4 complex yielded a map with an overall resolution of 2.95 Å (Supplementary Fig. 4), displaying excellent side-chain densities (Supplementary Fig. 4e), which enabled the assignment of most regions of hMRP4 (Fig. 2a).

The PGE1-bound hMRP4 structure is highly similar to the nucleotide-free hMRP4 structure (root-mean-square deviation [RMSD] = 0.96 Å), suggesting that substrate binding did not induce notable conformational rearrangement and the side-chain orientation of hMRP4 (Supplementary Fig. 5a), unlike bMRP1 in which LTC_4_ binding induced the two TM bundles to move closer to each other (Supplementary Fig. 5b). In the cryo-EM map of PGE1-bound hMRP4, we observed an extra EM density inside the binding pocket formed by TM3, TM5, TM6, and TM12, which could reasonably represent a PGE1 molecule (Fig. 2b, c). To confirm that the extra EM density was actually derived from PGE1, we performed independent explicit-solvent all-atom molecular dynamics (MD) simulations which suggested that PGE1 was stably bound in the binding pocket (Supplementary Fig. 5c). The structural analysis demonstrated that residues, including F211, F324, L363, L367, F368, M990, G991, W995, and R998, play an essential role in shaping the configuration of the binding pocket (Fig. 2c), a major part of which contains hydrophobic side chain, indicating hydrophobic interactions played essential roles in stabilizing the PGE1 substrate in the binding pocket (Figs. 2b, c). PGE1 comprises a cyclopentenone ring and two hydrophobic tails, with one containing a carboxylic acid group and the other bearing a hydroxyl group. Notably, the cyclopentenone ring of PGE1 was stabilized by the hydrophobic surface of F324 and W995; meanwhile, the two hydrophobic tails of PGE1 were inserted deeply into the hydrophobic pocket (H pocket). The carboxylic acid-bearing tail interacted with the positively charged R998 through electrostatic interactions. Collectively, the modeling suggested that PGE1 was trapped in the hMRP4 binding pocket via a combination of different interactions.

**Fig. 3:**
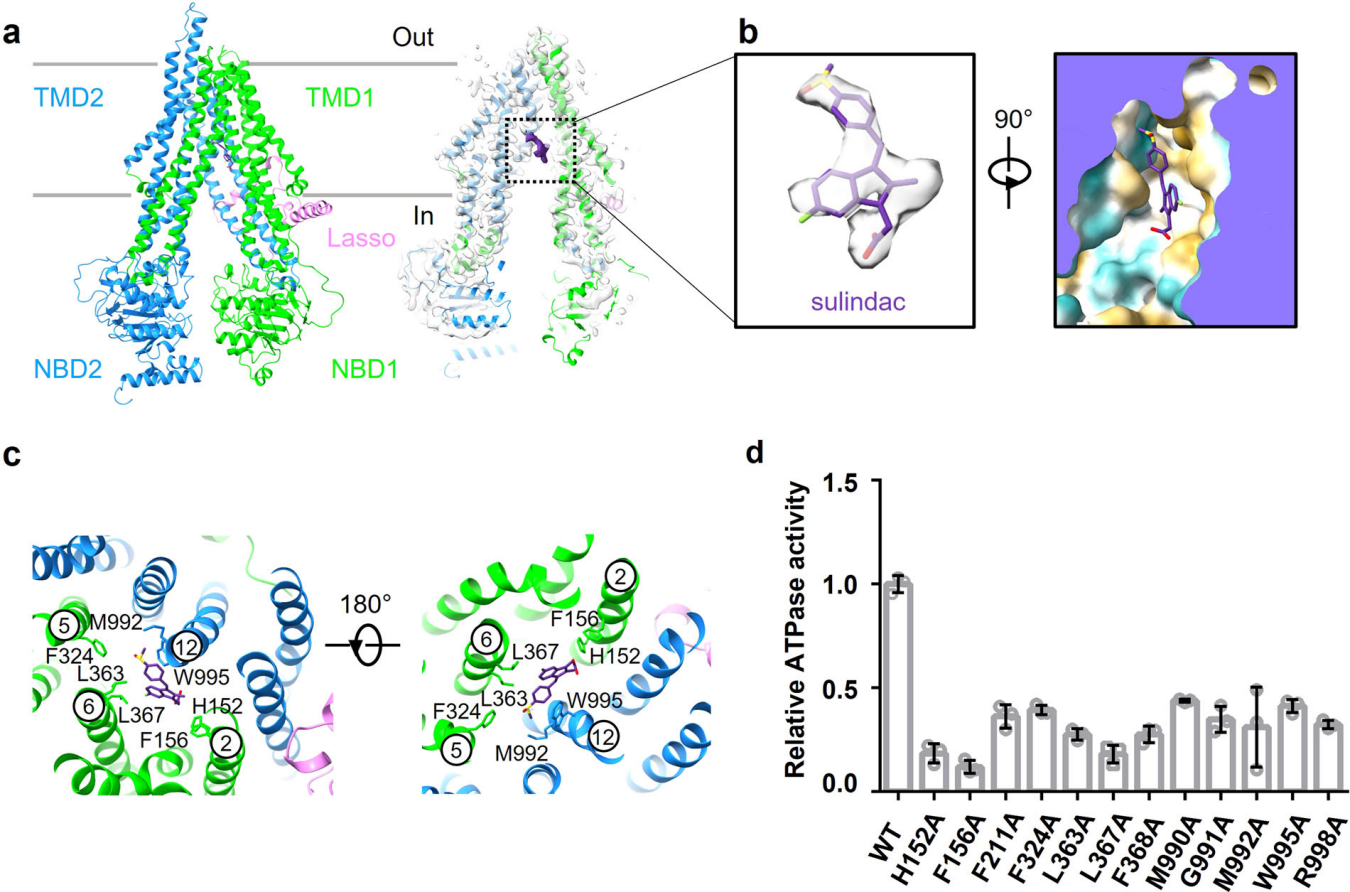
Structural features of sulindac-bound hMRP4. **a** Cartoon representation of the atomic model of sulindac-bound hMRP4 (left). Cryogenic electron microscopy map of sulindac-bound hMRP4 (right). **b** Electron microscopy density of sulindac in the same orientation as (A) (left). Sulindac is inserted into a pocket formed by the hydrophobic residues of hMRP4 (right). Contour level is 0.424. **c** Coordination of sulindac by hMRP4. Residues related to substrate binding are shown as sticks. Transmembrane helices (TMs) 2, 5, 6, and 12, which interact with sulindac, are labeled. **d** Relative ATPase activities of hMRP4 and mutants in a detergent of n-dodecyl-β-D-maltoside and cholesteryl hemisuccinate with 2 mM ATP in the absence of substrate (PGE1) or inhibitor (sulindac). Each data point is the mean of three independent experiments (*n* = 3), and the error bars represent the mean ± standard deviation. Source data are provided in Supplementary Data 3.

Next, to validate the importance of PGE1-associated residues, we introduced mutations in PGE1 binding pocket and examined their effects on the ATPase activity of the hMRP4 variants. Compared with the wt-hMRP4, all hMRP4 variants showed similar protein expression levels (Supplementary Fig. 1c) but displayed notably lower levels of ATPase activity (Fig. 3d), suggesting that these residues could directly influence the ATPase activity of hMRP4 and might impact the transport of PGE1.

### The inward-open structure of inhibitor-bound hMRP4

The PGE1-bound structure of hMRP4 provided a structural basis for understanding the mechanism of substrate transport. To further understand the mechanism of hMRP4 inhibition, we sought to determine the inhibitor-bound structures by incubating hMRP4 with different inhibitors, including indomethacin, MK571, quercetin, and sulindac. Finally, we resolved a cryo-EM structure of hMRP4 in complex with sulindac with an overall resolution of 3.77 Å (Fig. 3 and Supplementary Fig. 6), which showed excellent side-chain densities (Supplementary Fig. 6e), allowing us to trace most regions of the complex and the bound sulindac (Fig. 3a).

An EM density corresponding to sulindac was clearly defined inside a binding pocket formed by TM2, TM5, TM6, and TM12 (Fig. 3b, c). Sulindac was in an extended conformation and trapped in the pocket formed by a large number of hydrophobic residues, including F156, F324, L363, L367, G991, M992, and W995. The methylsulfinyl-benzylidene ring moiety was anchored to the hydrophobic surface formed by F324, L363, G991, M992, and W995. The -SOCH_3_ functional group in the benzene ring was in contact with F324 and L363, and the benzene ring interacted with W995 through π-π stacking interactions. The fluoromethyl-indene ring was located in the pocket formed by H152, F156, and L367. As for PGE1, we also assessed the cryo-EM model of the sulindac-bound hMRP4 complex by all-atom MD simulations, which suggested that sulindac was stable in the binding pocket of hMRP4 (Supplementary Fig. 5c).

We next assayed the ATPase activity of hMRP4 variants with single point mutations in the pocket-associated residues, including H152A, F156A, F324A, L363A, L367A, G991A, M992A, and W995A. We found that these mutations did not affect the protein expression of hMRP4 (Supplementary Fig. 1c); however, hMRP4 mutants exhibited lower ATPase activity than wt-hMRP4 (Fig. 3d), indicating that these residues directly influence the ATPase activity of hMRP4. Collectively, the cryo-EM structures, MD simulations, and biochemical results strongly supported that sulindac-associated residues play an important role in regulating the activity of hMRP4.

In addition, sequence alignment revealed that all of the MRP4 pocket-related residues are highly conserved across different species (Supplementary Fig. 7a), suggesting the transport mechanism of MRP4 may be evolutionarily conserved.

### Sulindac competes with PGE1 for the same binding pocket

**Fig. 4:**
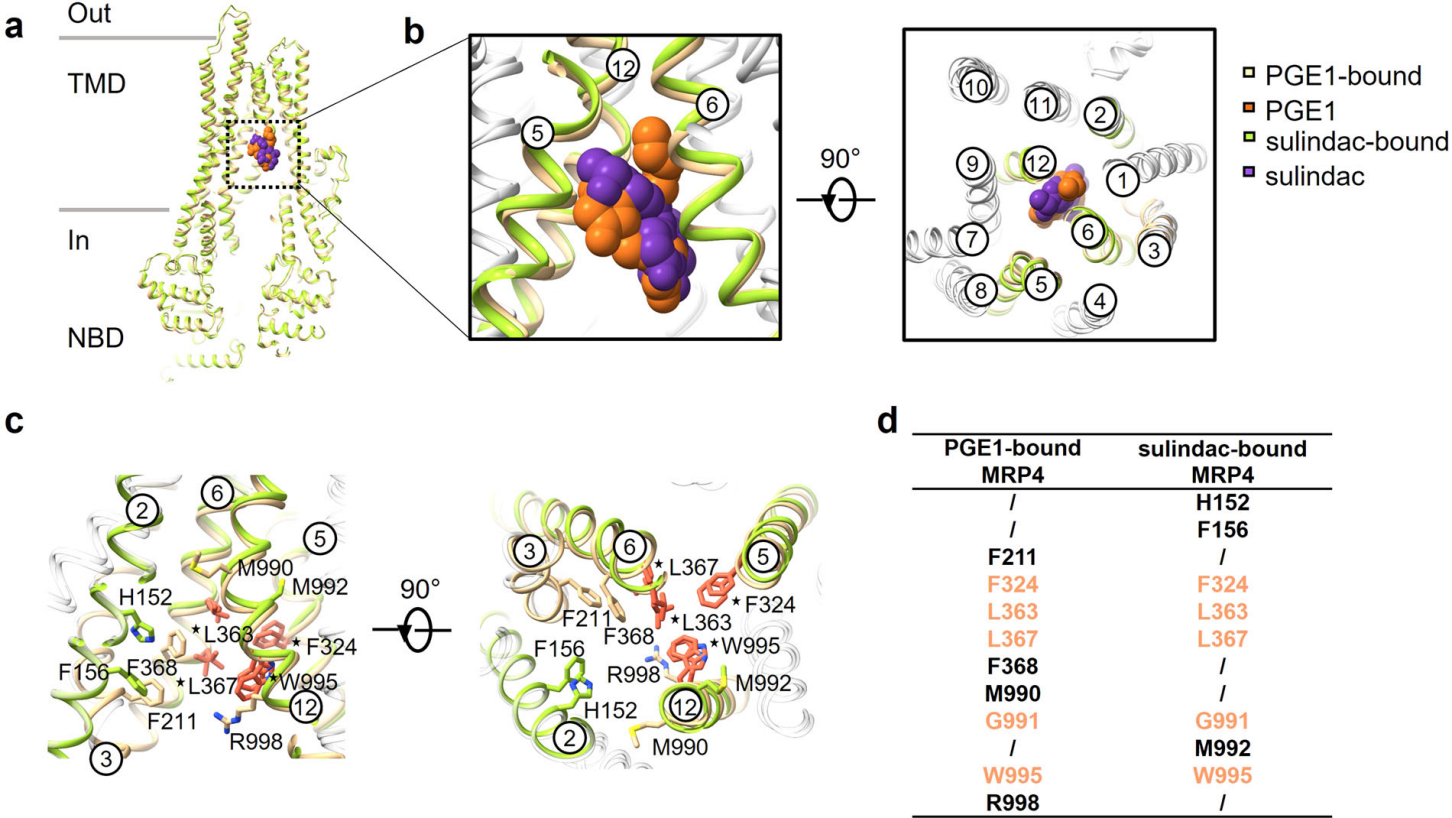
Comparison of prostaglandin E1- and sulindac-bound hMRP4 structures. **a** Cartoon representation of the overlap of prostaglandin E1 (PGE1)- (burly wood) and sulindac-bound (yellow-green) hMRP4. PGE1 and sulindac are shown in sphere representation and colored in chocolate and rebecca purple, respectively. **b** Closeup view of the PGE1- and sulindac-binding pockets. The shared binding transmembrane helices (TMs) of PGE1 and sulindac, including TM5, 6, and 12, are labeled (left). Extracellular view of hMRP4 (right). The twelve TMs of hMRP4 are numbered. **c** Overlap of binding sites of PGE1 and sulindac. Residues related to PGE1 or sulindac binding are shown in burly wood and yellow-green, respectively. The stars indicate the binding sites shared by the substrate and inhibitor; the residues are colored in salmon. The ligands are omitted for visual clarity. **d** Table featuring the substrate- and inhibitor-binding sites. The shared binding sites are colored in salmon.

Based on the PGE1-bound and sulindac-bound structures of hMRP4, we further investigated the mechanism of substrate transport blocking by the inhibitor. Structural superimposition suggested that both hMRP4 structures shared a highly similar inward-open conformation with an RMSD of 1.09 Å (Fig. 4a). A close-up view of the binding pocket revealed that the residues, comprising the PGE1 binding pocket, mainly came from TM3, TM5, TM6, and TM12 (Fig. 2c), whereas the sulindac binding pocket was formed mainly by TM2, TM5, TM6, and TM12 (Fig. 3c). Moreover, the two ligand-binding pockets are partially overlapped (Fig. 4b, c), suggesting that the inhibitor sulindac competes with PGE1 for part of the binding pocket although not with identical residues (Fig. 4d). The hydrophobic residues, including F324 (TM5), L363 (TM6), L367 (TM6), and G991 and W995 (TM12), are important to the binding of both PGE1 and sulindac. However, R998 (TM12) and F211 (TM3) contribute more to the binding of PGE1 than sulindac which was instead stabilized by H152 and F156 (TM2) (Fig. 4c). In addition, M990 contributes to the interaction with PGE1, but M992 with sulindac.

Interestingly, in the MD simulations of the PGE1-bound and sulindac-bound inward-open hMRP4 structures, we observed NBD1 and NBD2 were approaching, resulting in a quicker domain closure in the PGE1-bound state than the sulindac-bound state (Supplementary Fig. 5d). The substrate-induced domain closure was also observed in bMRP1^23^. Based on the observation that hMRP4 exhibited higher ATPase activity in the presence of PGE1 than sulindac, it would be interesting to explore the potential connection between domain movement, conformational cycle, and ATPase activity.

Together, our results suggest that sulindac may inhibit the transport activity of hMRP4 by directly competing for the substrate-binding pocket, resulting in an inhibition model of hMRP4 similar to that of P-glycoprotein^31^.

### The outward-open structure of ATP-bound hMRP4

**Fig. 5:**
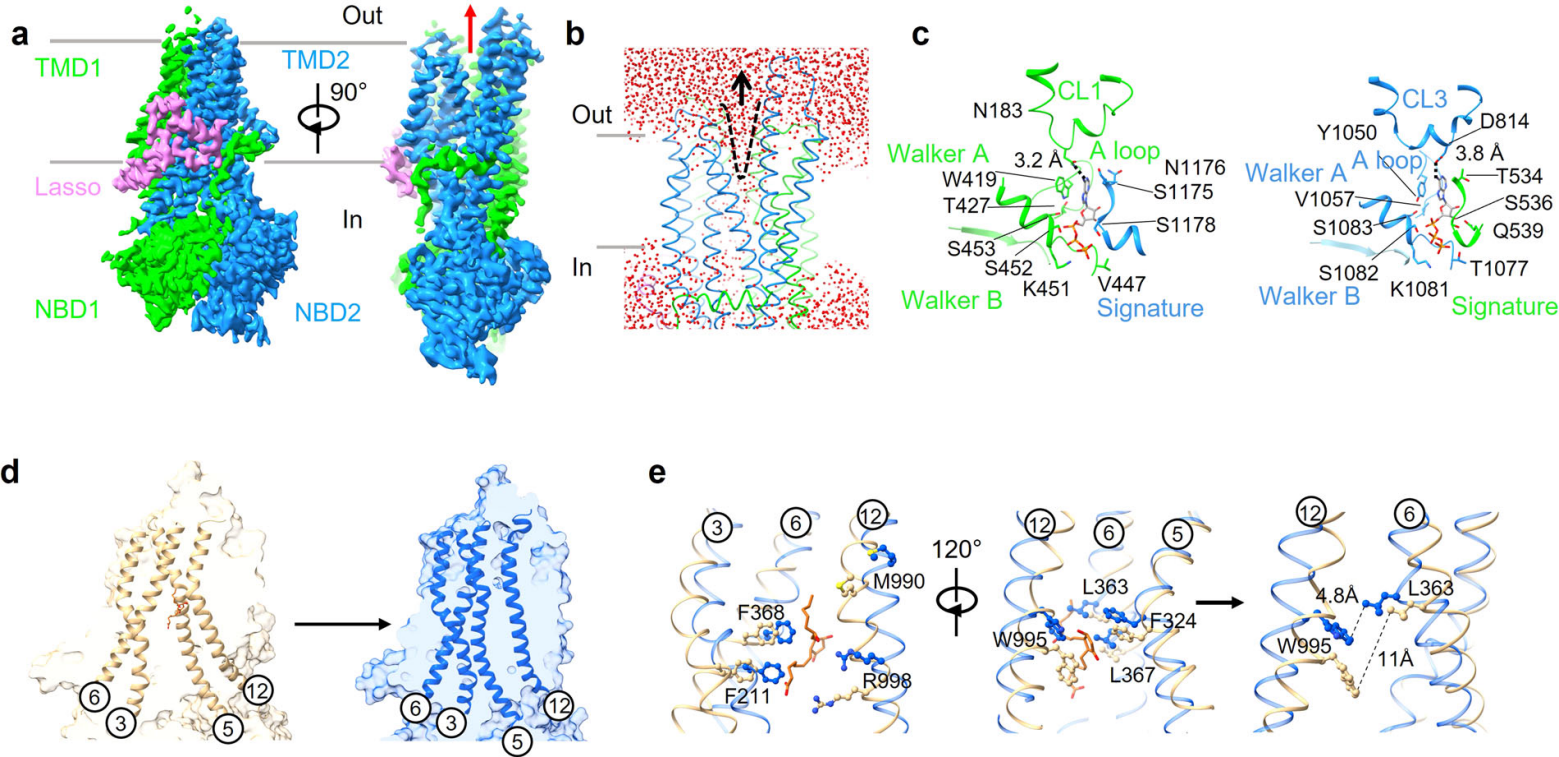
Structure of ATP-bound hMRP4 and comparison with the prostaglandin E1-bound hMRP4 structure. **a** Cryogenic electron microscopy map of ATP-bound hMRP4 (left). Two perpendicular side views are shown. The lasso domain is colored violet and the two halves of hMRP4 are colored lime and dodger blue for half 1 (transmembrane domain [TMD] 1 and nucleotide-binding domain [NBD] 1) and half 2 (TMD2 and NBD2), respectively. The red arrow indicates the conformational change upon ATP binding (right). Contour levels are 0.118 (lasso domain), 0.113 (TMD1 and NBD1) and 0.104 (TMD2 and NBD2). **b** The molecular dynamics simulations of the ATP-bound hMRP4. The water molecules are colored in red. **c** Close-up views of the degenerate (left) and consensus (right) ATPase sites. Interacting motifs of hMRP4 are shown in cartoon form with select side chains represented by sticks. ATP is represented by gray sticks with colored heteroatoms. **d** Conformational changes upon ATP binding accompanied with substrate release, as shown in cartoon and surface forms. The PGE1-bound inward-open and ATP-bound outward-open forms are colored in burly wood and blue, respectively. PGE1 is shown as sticks and colored chocolate. **e** Close-up view of the binding residues of the PGE1- and ATP-bound complexes viewed from within the plane of the membrane. The cartoon representation of select transmembrane helices shows select substrate-binding residues represented by sticks colored burly wood for the PGE1-bound structure and blue for the ATP-bound structure (left and middle). The distances between the tryptophan of TM12 and the leucine of TM6 are indicated (right).

To understand the substrate release pathway of hMRP4, we sought to obtain an outward-open hMRP4 conformation by capturing the nucleotide-bound state. Thus, hMRP4[EQ] was purified in the presence of 6 mM ATP-Mg^2+^ and subsequently subjected to single-particle cryo-EM analysis. We found that a majority of particles adopted a closed NBD conformation based on two-dimensional (2D) class averages (Supplementary Fig. 8). We obtained a well-defined three-dimensional (3D) electron microscopy map with an overall resolution of ~3.5 Å (Fig. 5a, Supplementary Fig. 8), enabling us to build the atomic model for most regions of hMRP4.

The structural comparison revealed that the structure of the ATP-bound hMRP4 presented a conformation resembling that of the outward-open ATP-bound bMRP1 and ABCD1 rather than the outward-occluded human SUR1 and ABCB6 (hSUR1) (Supplementary Fig. 9a, b). The MD simulations of the ATP-bound hMRP4 structure revealed a highly hydrated cavity formed inside the TM regions, which was not observed in the simulations of substrate- and inhibitor-bound hMRP4 (Figs. 2a, 3a). The hydrated cavity in the ATP-bound hMRP4 structure was exposed to the solvent and open to the outer leaflet, thus indicating that the ATP-bound structure we solved is likely in an outward-open state (Fig. 5b). Simulations of the substrate release process would provide further support, but the timescale is probably too long to be accessible to standard MD simulations although advanced enhanced sampling techniques would be helpful^32^.

The ATP-bound hMRP4 underwent an evident conformational change compared to the apo-hMRP4 (Supplementary Movie 1). The ATP binding induced the patching of TMDs by forming extensive contacts between the two halves of the transporter along the entire vertical axis. As a result, an obvious outward-open cavity was created by the curvature of the TMs, forming a substrate-releasing site (Fig. 5a). As expected, the NBDs formed a pseudo-symmetric “head-to-tail” dimer through the binding of ATP molecules, which were well-solved in the EM map, displaying clear densities corresponding to ATP and Mg^2+^ (Fig. 5c, Supplementary Fig. 8e). The NBDs interacted with ATP in a nearly identical manner even though one of them is a degenerate site and the other is a consensus site. The ATP molecules were stabilized by a number of highly conserved motifs among the MRP subfamily (Supplementary Fig. 7b), including the Walker A motif interacting with the ATP phosphate group and the A loop interacting with the ATP adenine group via π-π interactions (Fig. 5c). The ATP molecules also interacted with the signature motif through both -NH_2_ and phosphate groups. N183 of cytoplasmic loop 1 (CL1) and D814 of cytoplasmic loop 3 (CL3) appeared to interact with the -NH_2_ group over a 4 Å distance (Fig. 5c). In summary, the ATP molecule was captured in the NBDs of hMRP4 through a conserved ABC transporter binding model, and the outward-open hMRP4 conformation provided a structural basis for substrate release.

### Major conformational changes during substrate translocation

The global and local conformational changes of these structures provided a structural basis for hMRP4 substrate transport and inhibitor blocking of the transport process. Here, we captured four distinct states of hMRP4 along the ATP-driven transport cycle, including the apo inward-open (no substrate or ATP) (Fig. 1), substrate-bound inward-open (no ATP) (Fig. 2), inhibitor-bound inward-open (no ATP) (Fig. 3) and ATP-bound outward-open (no substrate, but ATP-Mg^2+^) (Fig. 5) states.

Superimposition of the PGE1-bound and ATP-bound structures revealed that ATP binding induced notable conformational rearrangements in the TM regions, among which the curvature of TM3, TM5, TM6, and TM12 plays essential roles in the formation of an outward-open pocket and substrate extrusion from the plasma membrane (Fig. 5d). The binding pocket-associated residues, especially F211, F324, M990, and R998, move relative to one another during the transition from the nucleotide-free structure to the nucleotide-bound structure (Fig. 5e), resulting in the collapse of the substrate-binding pocket. Consequently, the binding pocket loses its binding affinity for PGE1. The conformational rearrangement of the binding pocket effectively pushes PGE1 to the extracellular region through the outward-open pocket along the translocation pathway (Fig. 5d). These local conformational rearrangements all contribute to translocation to the extracellular space.

## Discussion

For ABC transporters, several models have been proposed to clarify how substrates are translocated and released outside the membrane and how inhibitors block these functions^28,30,31,33–35^. Previous models suggested that the energy of ATP hydrolysis is used to break the NBD interactions, subsequently, recover the conformation of ABC transporters to the inward-facing states for starting a new transport cycle^33,34^. Inhibitors with a high binding affinity were thought to accelerate ATP hydrolysis, thereby inducing the relative movement of the NBDs and closing the inward-facing pocket, but at the same time inhibit the transport process by blocking the substrate transport pathway^33^.

**Fig. 6:**
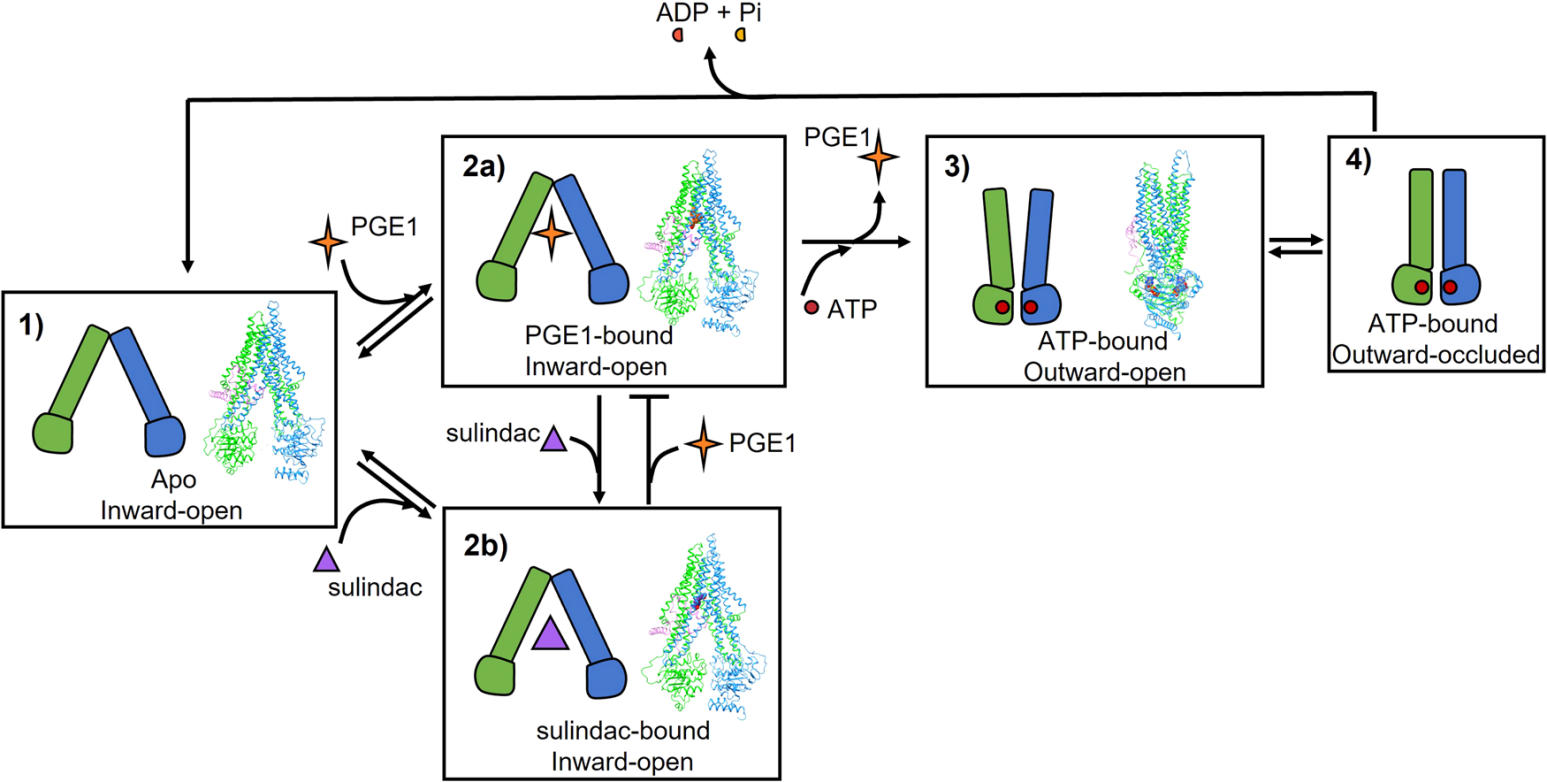
Proposed mechanism of hMRP4 substrate transport and small-molecule inhibition. Schematic of the proposed hMRP4 transport cycle in the presence of substrate (PGE1, orange star) and inhibitor (sulindac, purple triangle). ATP is shown as a red circle. Major conformational states are represented by numbers. State 1: apo. States 2a and 2b: PGE1- and sulindac-bound. State 3: outward-open conformation upon ATP-bound. State 4: outward-occluded conformation upon ATP-bound.

Here, we propose a model of the transport mechanism of hMRP4 based on our high-resolution cryo-EM structures (Fig. 6). We show that, in the absence of ATP and ligand (step 1), hMRP4 rests in an inward-open state in which the NBDs are widely separated, with the substrate-binding pocket accessible to the solvent in the cytoplasm. Both PGE1 and sulindac can be recruited and compete for the same ligand-binding pocket (steps 2a and 2b). The ligand binding only induces a remarkable conformational change of TMDs and NBDs once ATP molecules are recruited to the NBDs. ATP binding leads to a pronounced conformational rearrangement of the TM region, yielding an outward-open cavity. Meanwhile, the movement of TM helices squeezes and destroys the PGE1-binding pocket, resulting in the efflux of PGE1 from the cell (step 3) and subsequently yielding an ATP-bound outward-occluded state (step 4). However, sulindac may not be able to extrude by hMRP4 but keep binding in the substrate pocket due to its high binding affinity, resulting in the inhibition of hMRP4 (step 2b). After ATP hydrolysis, the dissociation of ADP and inorganic phosphate would reset the transporter to a resting state, ready for a new transport cycle.

We attempted to capture the ATP-bound state of hMRP4 using the EQ mutation, but it was challenging to collect enough NBD-dimerized particles of hMRP4[EQ] mutant for 3D reconstruction, indicating that physiologically dimerized NBDs tend to be transient and remain flexible even in 6 mM ATP and Mg^2+^. Therefore, to collect sufficient NBD-dimerized hMRP4 particles for 3D EM reconstruction, besides the engineered EQ mutation, we also introduced two cysteine mutations in each NBD (E568C and Q1275C) to stabilize the dimerized NBDs of hMRP4 and added 2 mM ATP-Mg^2+^ in the whole hMRP4 purification process. However, the EM density corresponding to the disulfide bond was not observed in the ATP-bound hMRP4 map, indicating the dimerized NBDs were mainly stabilized by the binding of ATP molecules, as has been reported for other ATP-bound ABC transporters^22,36,37^.

We note that during the preparation of our manuscript, several hMRP4 structures were reported in the substrate-bound and inhibitor-bound states^38–40^. Interestingly, in these studies, the ATP-bound structures were in the outward-occluded conformations^39,40^, different from the outward-open conformation in our ATP-bound hMRP4 and the reported ATP-bound bMRP1 structures solved in detergent^22^. The conformational difference might be caused by several potential factors, including the expression systems, purification processes, and reconstituted conditions. In these works, hMRP4 proteins were expressed in sf9 cells and reconstituted with nanodisc^39,40^, but the counter proteins were purified from mammalian cells and solved in digitonin in our work. Therefore, the ATP-bound outward-open structure we captured, together with the apo inward-open, substrate-bound inward-open, and inhibitor-bound inward-open structures, would provide valuable information on the transport mechanism of hMRP4.

One of the preprint drafts described the conformational changes of hMRP4 after binding the substrate^39,40^. However, the substrate-bound and apo- structures of our hMRP4 have almost the same conformation, consistent with the observation in another preprint work of hMRP4 available on BioRxiv^38–40^. Interestingly, the conformational rearrangements of hMRP4 were different in two preprint studies even though the same PGE2 substrate was added. Therefore, we believe some uncertain factors impact the conformation changes of hMRP4, which awaits further studies.

Moreover, in hMRP4, both PGE1 and sulindac were captured in a hydrophobic pocket formed by residues mainly from TM5, TM6, and TM12; however, in bMRP1, LTC_4_ was trapped in the H pocket comprising residues mainly from TM10, TM14, and TM17 (the counterpart transmembrane helices were TM5, TM9, and TM12 in hMRP4) (Supplementary Fig. 4c). Therefore, the substrate binding model might be different in distinct MRP proteins. In addition, the structural analysis revealed that several residues contribute to PGE1 and sulindac binding, and follow-up biochemical assays demonstrated that most mutation variants had no apparent effect on hMRP4 ATPase activity. However, testing the ATPase activity of hMRP4 variants in the presence of PGE1 or sulindac is still needed, which could give more evidence of the importance of these ligand-associated residues.

The various substrates and inhibitors reportedly recognized by hMRP4 can be divided into fat-soluble and water-soluble groups. Both PGE1 and sulindac are fat-soluble and were trapped in hMRP4 mainly through hydrophobic forces; therefore, the basis of PGE1 and sulindac binding deduced from our solved hMRP4 structures may prove useful for understanding the basis of binding other fat-soluble ligands, such as methotrexate^41^. For water-soluble ligands, such as cGMP and cAMP^7^, the substrate recognition mechanism might be different.

hMRPs are putative drug targets for the treatment of cancer^42^. However, to date, only structures of bMRP1 in the MRP subfamily have been reported^22,23^, which is insufficient to understand the binding mechanisms of human MRPs. Our cryo-EM structures of hMRP4 in complex with different ligands, especially the inhibitor sulindac, provide a structural basis for the rational development of drugs targeting hMRP4. Moreover, our well-solved structures of hMRP4 may be helpful for elucidating the transport mechanisms of other MRP proteins.

Collectively, our findings provide direct structural evidence of sulindac-mediated inhibition of PGE1 transport by hMRP4. Our work sheds light on the molecular basis of hMRP4 ligand recognition and transport and establishes a framework for future biophysical studies and structure-based drug design.

## Methods

### Protein expression and purification

The codon-optimized full-length human *ABCC4* gene encoding the MRP4 protein (UniProt ID: O15439) was cloned into the pCAG vector with the C-terminal 3×FLAG epitope tags (DYKDHDGDYKDHDIDYKDDDDK) using homologous recombination with a ClonExpress Ultra One Step Cloning Kit (Vazyme Biotech). Site-directed mutagenesis was performed with the Fast Mutagenesis System kit (Transgen) using a standard two-step PCR and verified by DNA sequencing (Shenggon Biotech, Shanghai). The plasmids and primers information are provided in Supplementary Data 1 and 2.

For protein expression, HEK293F cells were cultured in SMM 293-TII medium (M293TII, Sino Biological) in the following conditions: 37 °C and 5% CO_2_ at 110 rpm in a shaker. When the cell density reached ~2.0 × 10^6^ cells mL^−1^, ~1.5 mg plasmids were transfected into 800 mL HEK293F cells with 4.5 mg linear polyethylenimine (Polysciences); each 800 mL cells was pre-incubated in 40 mL fresh medium for 20 min, which was then added to the cells. The transfected cells were grown at 37 °C for 16 h. Afterwards, 10 mM sodium butyrate (Sigma-Aldrich) was added and the cells were cultivated at 30 °C for an additional 48 h prior to harvest. After centrifugation at 2000 rpm for 5 min, the cell pellets were resuspended and washed with 1×PBS, quickly frozen in liquid nitrogen, and stored at −80 °C until further use.

For purification, the following steps were performed at 4 °C. Cell pellets were thawed and gently resuspended in lysis buffer containing 100 mM Tris-HCl (pH 8.0), 150 mM NaCl, 1 mM phenylmethylsulfonyl fluoride (PMSF), 2 mM Dithiothreitol (DTT), 1% (w/v), n-dodecyl-β-D-maltoside (DDM) (Anatrace), and 0.2% (w/v) cholesteryl hemisuccinate CHS (Sigma) with EDTA-free protease inhibitor cocktail (Roche). Two hours later, the cell lysate was subjected to centrifugation at 20,000 rpm for 1 h and the supernatant was transferred to an anti-FLAG M2 affinity gel (Sigma-Aldrich), which was rotated at 4 °C for 1.5 h. Then, the resin was collected and rinsed with 20 column-volumes of wash buffer containing 25 mM Tris-HCl (pH 8.0), 150 mM NaCl, 10% glycerol (v/v), 2 mM DTT, and 0.06% digitonin (w/v) (BID3301, Apollo Scientific). The protein was eluted with an elution buffer containing 25 mM Tris-HCl (pH 8.0), 150 mM NaCl, 5% glycerol (v/v), 2 mM DTT, and 0.06% digitonin (w/v) supplemented with 200 μg mL^−1^ FLAG peptide. The protein eluent was concentrated using a 100-kDa cut-off Centricon (Millipore) and further purified by SEC using a Superose 6 Increase column (GE Healthcare) in an SEC buffer containing 25 mM Tris-HCl (pH 8.0,) 150 mM NaCl, 2 mM DTT, and 0.06% digitonin (w/v). Peak fractions were pooled and concentrated for further biochemical studies or cryo-EM experiments.

All mutant proteins used in this project were expressed and purified using the same protocol as that described for the wt protein.

### ATPase activity assay

The ATPase activities of hMRP4 and its mutants were measured with a commercially available kit, based on measurement of the inorganic phosphate (Pi) released from ATP, according to the kit protocol (Nanjing Jiancheng Bioengineering Institute, China) in 96-well plates at an optical density of 636 nm. To measure the ATPase activity of hMRP4 in the presence or absence of the substrate or inhibitor, or varying ATP concentrations, the hMRP4 proteins (final concentration: 0.03 μM) were added to the reaction buffer containing 25 mM Tris-HCl (pH 8.0), 150 mM NaCl, 2 mM DTT, 0.02% (w/v) DDM, 0.004% (w/v) CHS, and 2 mM MgCl_2_. Substrates or inhibitors were diluted to the required concentrations and added to the reaction mixture, which was incubated at 37 °C. ATP was supplemented at a final concentration of 2 mM to assay ATPase activity in the presence of different concentrations of substrate or inhibitor, or at the indicated concentration, to initiate the reaction, which occurred at 37 °C for 20 min. The reactions were mixed with matrix buffer, and the supernatant was collected after centrifugation at 3500 rpm for 10 min, and then further incubated with chromogenic agent and termination solution, sequentially. Finally, the amount of released Pi was quantified.

In the substrate-stimulated or inhibitor-treated ATPase activity assays, all procedures were as described above, except that the final protein concentration was 0.1 μM and the reaction time was 45 min. Statistical analysis of the ATP activity assay results were fit by nonlinear regression to the Michaelis-Menten using GraphPad Prism. Data represent the mean ± standard deviation of three independent measurements.

### Parameters and protocols of MD simulations

The models of the PGE1-bound and sulindac-bound hMRP4 complexes in the inward-open state and ATP-bound outward-open state were constructed using the corresponding cryo-EM structures. The models were subsequently embedded in a flat, mixed lipid bilayer consisting of 1-palmitoyl-2-oleoyl-sn-glycero-3-phosphocholine, and solvated in a cubic water box containing 0.15 M NaCl. The size of the box was 11.8, 11.8, and 15.6 nm in the x, y, and z dimensions, respectively, resulting in ∼222,000 atoms in total for each model. The OPM (Orientations of Proteins in Membranes) webserver was used to align the TM region in the lipid bilayer. The systems were built with the CHARMM-GUI webserver^43^ and underwent an energy minimization step using the steepest descent algorithm, followed by a six-step equilibration during which position constraints in the systems were gradually removed. Finally, production runs in semi-isothermal-isobaric (NPT) conditions were performed using the CHARMM36 force field^44^ (CHARMM36m for proteins, CHARMM36 for lipids, and TIP3P for water) and the available CGenFF parameters^45^ for the PGE1, sulindac and ATP molecules. In the MD simulations, the temperature was kept constant at 310 K using a Nosé–Hoover thermostat with a 1-ps coupling constant, and the pressure at 1.0 bar using the Parrinello–Rahman barostat with a 5-ps time coupling constant. A cut-off of 1.2 nm was applied for the van der Waals interactions using a switch function starting at 1.0 nm. The cut-off for the short-range electrostatic interactions was also 1.2 nm, and the long-range electrostatic interactions were calculated by means of the particle mesh Ewald decomposition algorithm with a 0.12-nm mesh spacing. A reciprocal grid of 100 × 100 × 144 cells was used with fourth-order B-spline interpolation. All simulations were performed using a GPU-accelerated version of Gromacs 2021.5^46^. Two independent simulations (1000 ns for each) were performed for the PGE1-bound and sulindac-bound inward-open state. A 500 ns simulation was performed for the ATP-bound outward-open state. Trajectories were analyzed using PLUMED^47^.

### cryo-EM sample preparation and data acquisition

The purified hMRP4 proteins were concentrated to 5–10 mg mL^−1^ for further cryo-EM sample preparation. For the substrate PGE1-bound and inhibitor sulindac-bound complexes, the hMRP4 proteins were incubated with 100 µM PGE1 (Selleck Chemicals, Cat. no. S1508) and 80 µM sulindac (Selleck Chemicals, Cat. no. S2007), respectively, for 1 h on ice before sample vitrification. For the ATP-bound complex, the protein was incubated with 6 mM ATP-Mg^2+^ at room temperature for 1 h before being applied to the grids. For all cryo-EM samples, proteins (3.5 μL) were applied to a glow-discharged (0.39 mBar air, 15 mA, 50 s) holey carbon grid (Quantifoil R1.2/1.3, Au, 300 mesh), and subsequently vitrified using a FEI Vitrobot Mark IV (Thermo Fisher Scientific) set to 10 °C and 100% humidity. The grids were blotted using Whatman No. 1 filter paper for 4 s at 10 °C in 100% humidity and then plunge-frozen in liquid ethane. All grids were finally stored in liquid nitrogen for future data acquisition.

The prepared grids were transferred to a Titan Krios G3i microscope (Thermo Fisher Scientific), running at 300 kV and equipped with a Gatan Quantum-LS Energy Filter (GIF, slit width of 20 eV) and a Gatan K3 Summit direct electron detector in the super-resolution mode. Original movies were recorded with the following conditions: an accumulated dose of 50 electrons per Å^2^, a movie stack of 32 frames, a defocus value ranging from −1.2 to −1.8 μm, and a pixel size of 0.855 Å per pixel. For the sulindac-bound MRP4 complex, all movies were collected with a Titan Krios microscope equipped with a GIF-Quantum energy filter, operated at 300 kV, with a slit width of 20 eV, a nominal magnification of 96,000X (resulting in a calibrated physical pixel size of 0.86 Å/pixel) and a defocus range of −1.2 to −1.8 μm. All movies were recorded on a Falcon4 electron direct detector in the counted mode. A total dose of 50 electrons per Å^2^ was used, generating 32 frames. All the data acquisition was automatically collected using EPU software.

### Data processing

All movie stacks were aligned and motion-corrected using MotionCor2^48^, and the contrast transfer function (CTF) parameters were estimated using patch CTF estimation (cryoSPARC^49^). For all datasets, 100 micrographs were selected for automatic particle-picking using the Blob picker and subjected to 2D classification analysis. Good particles were selected to generate good particle templates for Topaz training. Then, all micrographs were picked by Topaz Extract using the trained Topaz model in cryoSPARC and extracted with a box of 300 pixels for all datasets. All 2D and 3D classifications and refinements were performed with cryoSPARC. The overall resolution of the final map was determined by the 0.143 criterion of the gold-standard Fourier shell correlation (FSC). The local resolution maps were evaluated by Local Resolution Estimation in cryoSPARC. The single-particle analysis procedures of all states are summarized in Supplementary information, Supplementary Figs. 6–9.

### Model building and refinement

A predicted atomic model was downloaded from the AlphaFold Protein Structure Database for initial atomic model building of all hMRP4 structures. Each hMRP4 model was first manually docked into the EM density maps with UCSF Chimera^49^. Subsequent model adjustments and rebuilding were done with Coot. The ligands were also fitted into the EM density map with Coot^50^ by obtaining ligand coordinates based on the 3-letter code and merging with the coordinates of hMRP4. Models were further refined against the cryo-EM density maps using Phenix (phenix.real_space_refine) with geometry restraints and secondary structure restraints imposed^51^. Structural figures were prepared using ChimeraX and PyMOL. The final refinement statistics are provided in Table 1.

**Table 1:** Overview of Cryo-EM data collection and coordinate refinement.

	Apo hMRP4	PGE1-bound hMRP4	ATP-bound hMRP4	Sulindac-bound hMRP4
Data collection and processing				
Magnification	105,000×	105,000×	105,000×	96,000×
Voltage (kV)	300	300	300	300
Camera	Gatan K3 Summit	Gatan K3 Summit	Gatan K3 Summit	Falcon4
Camera mode	Super-resolution	Super-resolution	Super-resolution	Super-resolution
Electron exposure (e^–^/Å^2^)	50	50	50	50
Defocus range (μm)	0.8–1.2	0.8–1.2	0.8–1.2	0.8–1.2
Pixel size (Å)	0.856	0.855	0.855	0.86
Symmetry imposed	C1	C1	C1	C1
Initial particle projections (no.)	1,854,905	2,147,528	389,704	371,999
Final particle projections (no.)	203, 347	378, 698	108,200	151,694
Map resolution (Å)	3.13	2.95	3.48	3.77
FSC threshold	0.143	0.143	0.143	0.143
Map resolution range (Å)	2.7–10	2.5– 8.5	2.3–9.7	1.95–9.45
Refinement				
Initial model used	Not applicable (N/A)	Not applicable (N/A)	Not applicable (N/A)	Not applicable (N/A)
Model resolution (Å)	3.13	2.95	3.48	3.77
FSC threshold	0.143	0.143	0.143	0.143
Map sharpening B factor (Å^2^)	−113.8	−133.8	−105.8	−144.2
Model composition				
Non-hydrogen atoms	9969	9917	10027	9858
Protein residues	1251	1301	1251	1237
Ligand		XPG	ATP MG	SUZ
R.m.s. deviations				
Bond lengths (Å)	0.011	0.011	0.012	0.005
Bond angles (°)	0.894	0.884	0.889	0.585
Validation				
MolProbity score	2.22	2.111	2.08	2.13
Clashscore	6	7	5	7
Rotamer outliers (%)	0.00	0.00	0.00	0.00
Ramachandran plot				
Favored (%)	98	98	98	95
Allowed (%)	2	2	2	5
Disallowed (%)	0.00	0.00	0.00	0.00

### Statistics and reproducibility

The sample sizes and statistical analyses used are presented in the legend of each figure.

### Reporting summary

Further information on research design is available in the Nature Portfolio Reporting Summary linked to this article.

## Supplementary information

Supplementary Information

Description of Additional Supplementary Files

Supplementary Data 1

Supplementary Data 2

Supplementary Data 3

Supplementary Movie 1

Reporting Summary

## Data Availability

The cryo-EM structures of apo-form hMRP4, PGE1-bound hMRP4, sulindac-bound hMRP4 and ATP-bound hMRP4 have been deposited at PDB under the codes of 8IZ8, 8IZ9, 8IZ7 and 8IZA, respectively. The cryo-EM density maps of three structures have been deposited at the Electron Microscopy Data Bank (EMD-35835, EMD-35836, EMD-35834 and EMD-35837, respectively).

## Code availability

Simulation input files and initial and final coordinate files for each simulation have been deposited in Github: https://github.com/yongwangCPH/papers/tree/main/2023/MRP4.
