# Supplementary material for: Structural basis for substrate and inhibitor recognition of human multidrug transporter MRP4
Source: Commun Biol. 2023 May 22;6:549. doi: 10.1038/s42003-023-04935-7 (PMC10202912; doi:10.1038/s42003-023-04935-7)
Supplement: Supplementary file 1 — Supplementary Information [file 42003_2023_4935_MOESM1_ESM.pdf]

Supplementary Figure 1

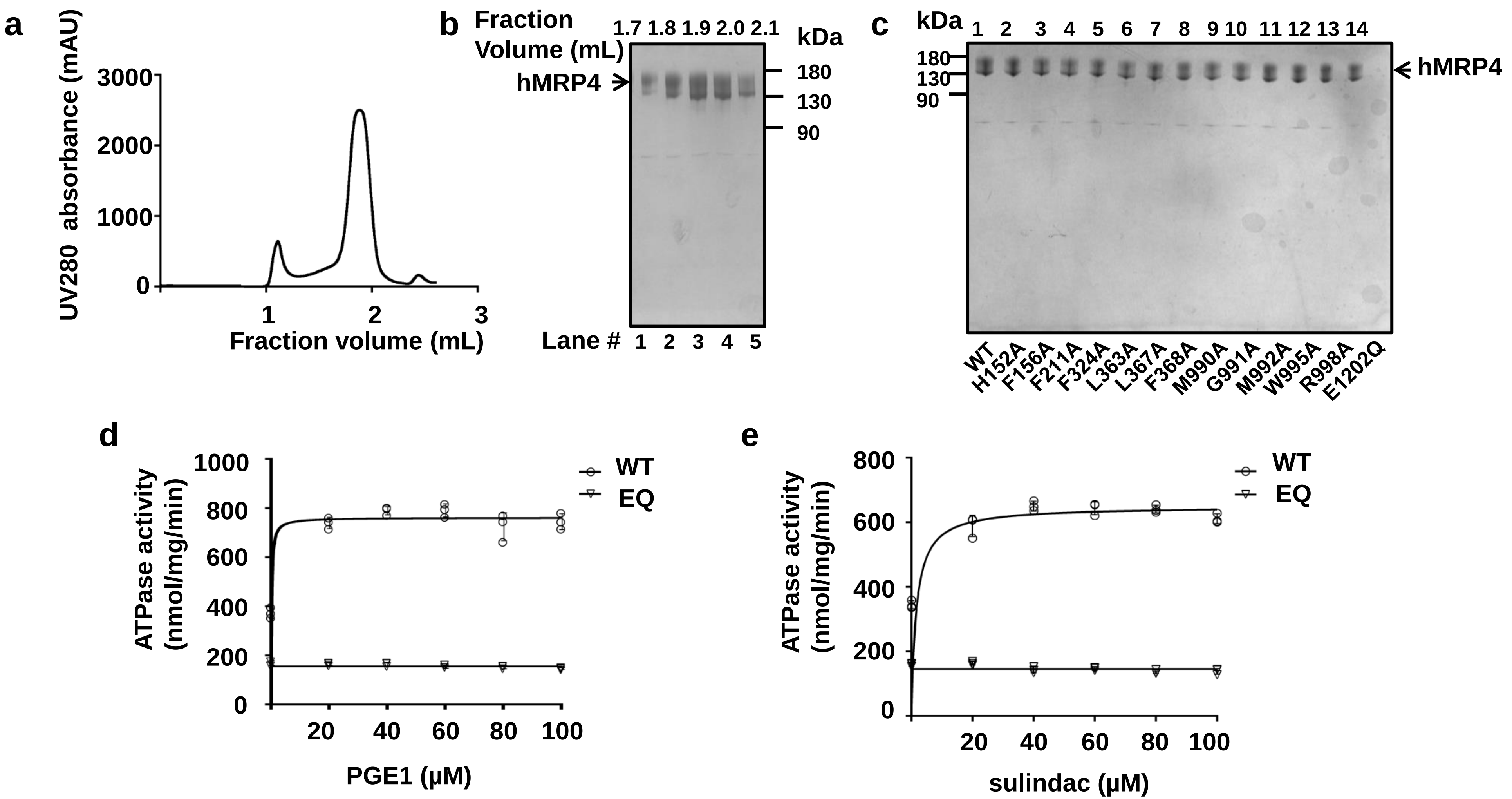

Supplementary Figure 1

Biochemical characterization of hMRP4.

a) The size exclusion chromatography profiles of hMRP4 using a Superose 6 Increase 5/150 GL SEC column.

b) The peak fractions around 1.9 mL were subjected to SDS-PAGE and visualized by Coomassie blue staining. The chromatogram and gel images are representative of >3 independent experiments with similar results.

c) Representative Coomassie blue-stained gel of wild-type (WT) hMRP4 and variants expressed in HEK293F cells and purified by Superose 6 Increase 5/150 GL SEC column. The data were reproduced in three independent experiments.

d) Prostaglandin E1 (PGE1) concentration-dependent ATPase activity of WT hMRP4 and E1202Q mutant hMRP4 upon addition of PGE1 in detergent containing n-dodecyl-β-D-maltoside and cholesteryl hemisuccinate. All data points represent the means of three independent measurements (n=3). Error bars indicate mean ± standard deviation. Lines are fitted by nonlinear regression of the Michaelies-Menten equation.

e) Sulindac concentration-dependent ATPase activity of WT hMRP4 and E1202Q mutant hMRP4 in detergent containing n-dodecyl-β-D-maltoside and cholesteryl hemisuccinate. All data points represent the means of three independent measurements (n=3). Error bars indicate mean ± standard deviation. Lines are fitted by nonlinear regression of the Michaelies-Menten equation.

Supplementary Figure 2

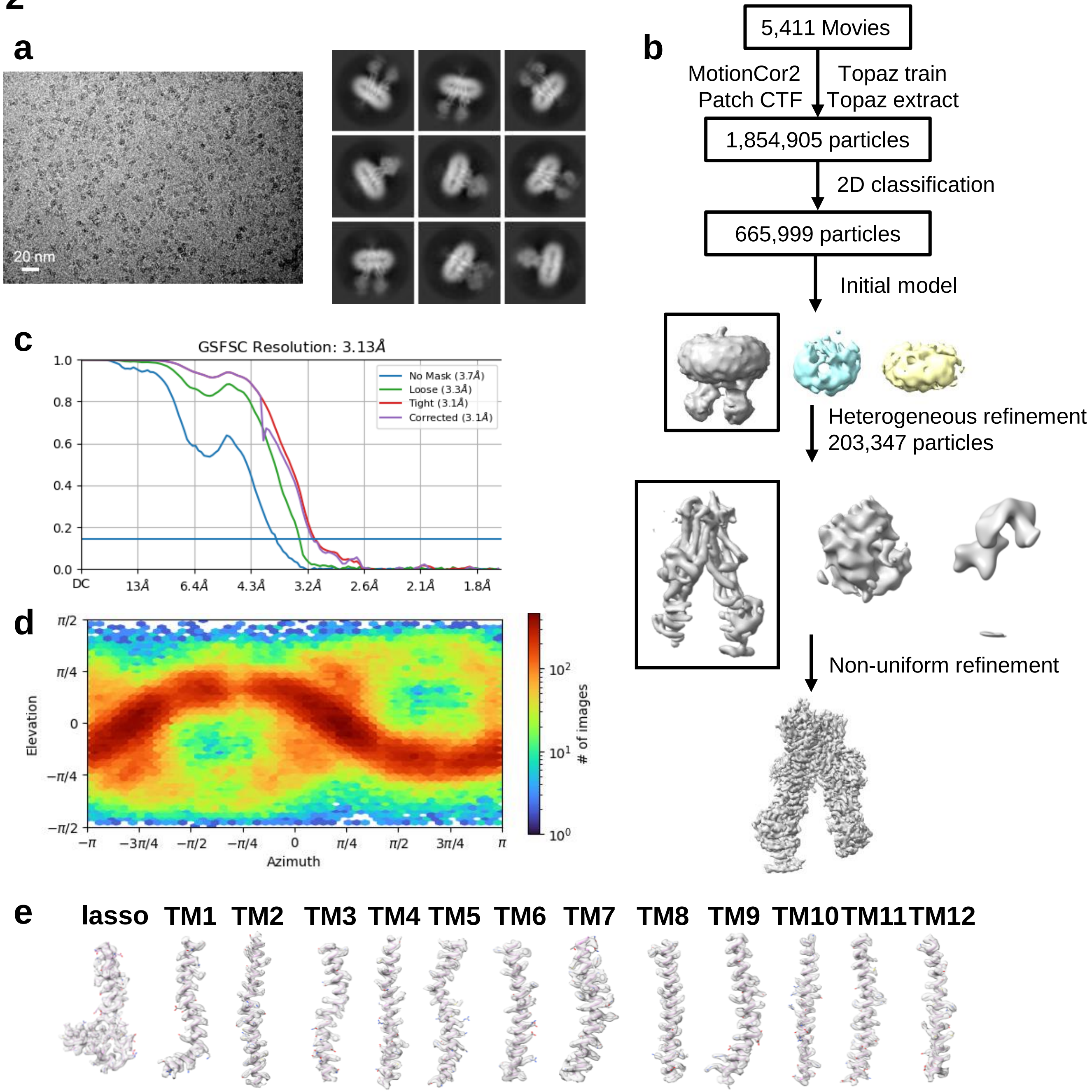

Supplementary Figure 2

Data processing and model building for apo hMRP4.

a) Representative cryogenic electron microscopy micrographs and two-dimensional (2D) averages. Bar: 20 nm. The micrograph is representative of 5,411 cryogenic electron microscopy images.

b) Flowchart for cryogenic electron microscopy data processing.

c) Gold-standard Fourier shell correlation (GSFSC) curve for the apo hMRP4 map generated using cryoSPARC 3.1.

d) Euler angle distribution of the classified particles used for the final three-dimensional refinement of the overall map.

e) Electron microscopy density of lasso domain and each transmembrane helix (TM) of apo hMRP4. Contour levels are 0.395 (lasso), 0.575 (TM1), 0.496 (TM2), 0.507 (TM3), 0.437 (TM4), 0.386 (TM5), 0.446 (TM6), 0.377 (TM7), 0.363 (TM8), 0.435 (TM9), 0.473 (TM10), 0.468 (TM11) and 0.385 (TM12).

Supplementary Figure 3

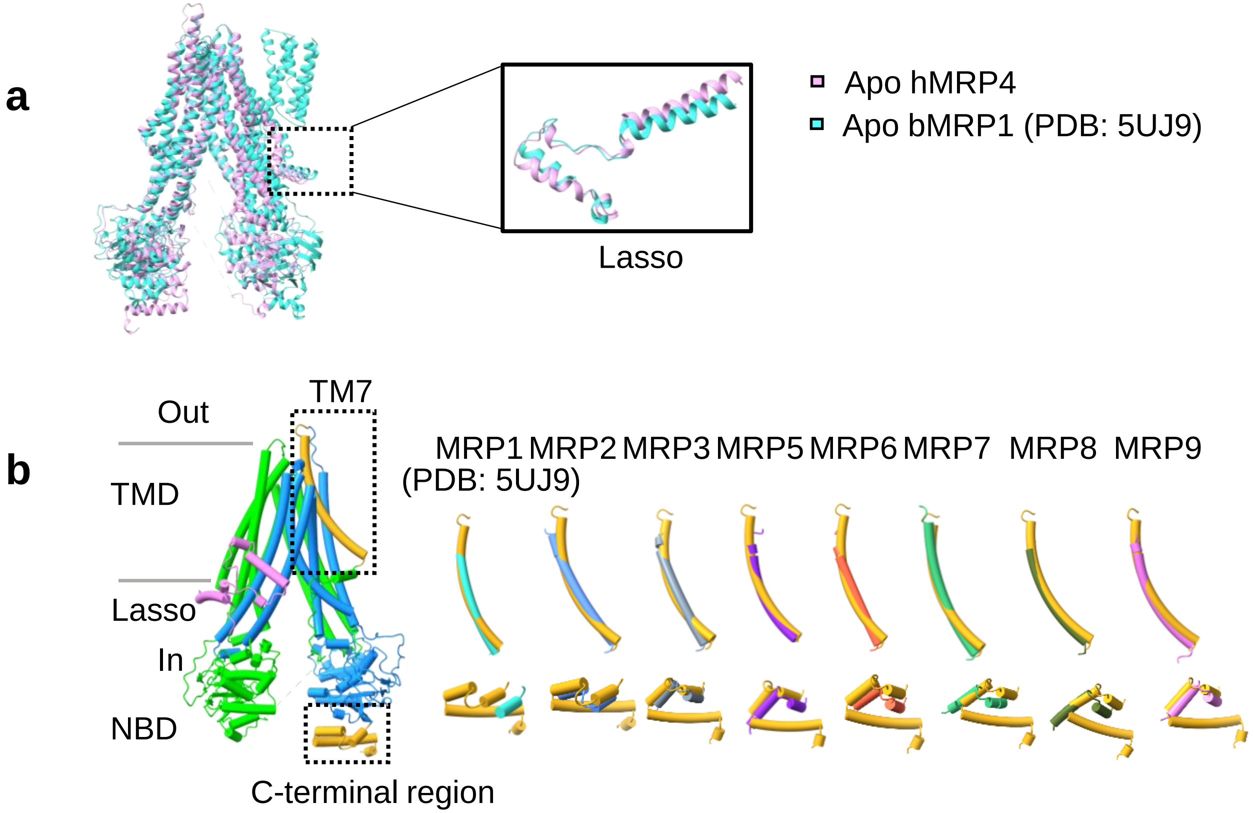

Supplementary Figure 3

Structural superimposition of hMRP4 on other MRP family members.

a) Comparison the strucutre of hMRP4 (pink) and bovine MRP1 (cyan) (PDB: 5UJ9).

b) Comparison of TM7 and C-terminal helices among hMRP4 and other MRP family members. The lasso domain is colored violet and the two halves of hMRP4 are colored in lime and dodger blue for half 1 (transmembrane domain [TMD] 1 and nucleotide-binding domain [NBD] 1) and half 2 (TMD2 and NBD2), respectively. In the cartoon form, TM7 and the C-terminal helices of hMRP4 are colored goldenrod. Unlike the structure of bovine MRP1 (PDB ID: 5UJ9), the structures of human MRP2, MRP3, MRP5, MRP6, MRP7, MRP8, and MRP9 were downloaded from AlphaFold Protein Structure Database (alphafold.ebi.ac.uk). The following proteins are pictured: bovine MRP1 (cyan), MRP2 (cornflower blue) MRP3 (light slate gray), MRP5 (blue violet), MRP6 (tomato), MRP7 (medium sea green), MRP8 (dark olive green), and MRP9 (orchid) are colored in cyan, cornflower blue, respectively.

Supplementary Figure 4

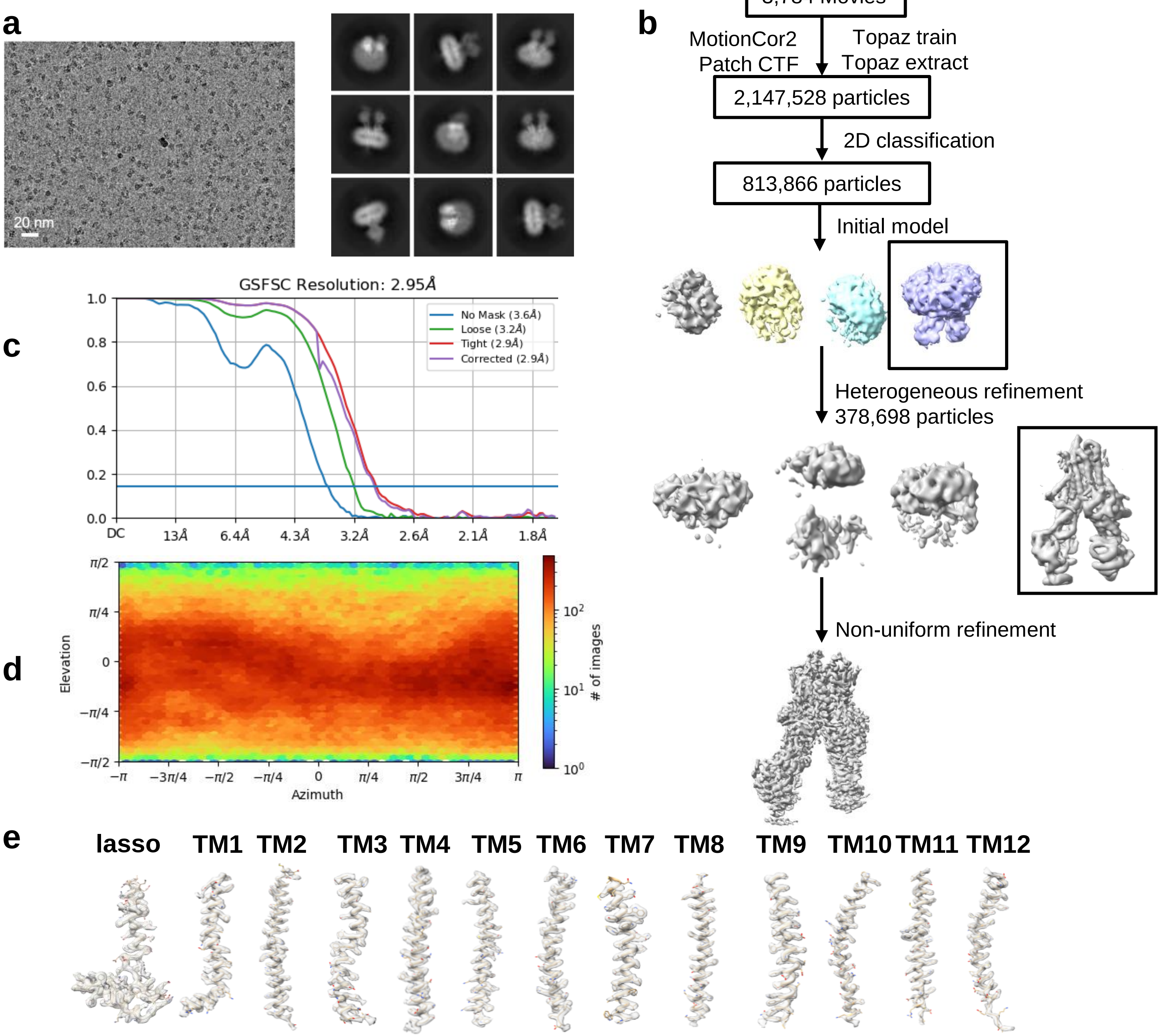

Supplementary Figure 4

Data processing and model building of prostaglandin E1-bound hMRP4.

a) Representative cryogenic electron microscopy micrographs and two-dimensional (2D) averages. Bar: 20 nm. The micrograph is a representative of 3,784 cryogenic electron microscopy images.

b) Flowchart for cryogenic electron microscopy data processing.

c) Gold-standard Fourier shell correlation (GSFSC) curve for the prostaglandin E1-bound hMRP4 map generated using cryoSPARC 3.1.

d) Euler angle distribution of the classified particles used for the final three-dimensional refinement of the overall map.

e) Electron microscopy density of lasso domain and each transmembrane helix (TM) of PGE1-bound hMRP4. Contour levels are 0.568 (lasso), 0.533 (TM1), 0.623 (TM2), 0.479 (TM3), 0.453 (TM4), 0.491 (TM5), 0.473 (TM6), 0.594 (TM7), 0.762 (TM8), 0.687 (TM9), 0.665 (TM10), 0.727 (TM11) and 0.66 (TM12).

Supplementary Figure 5

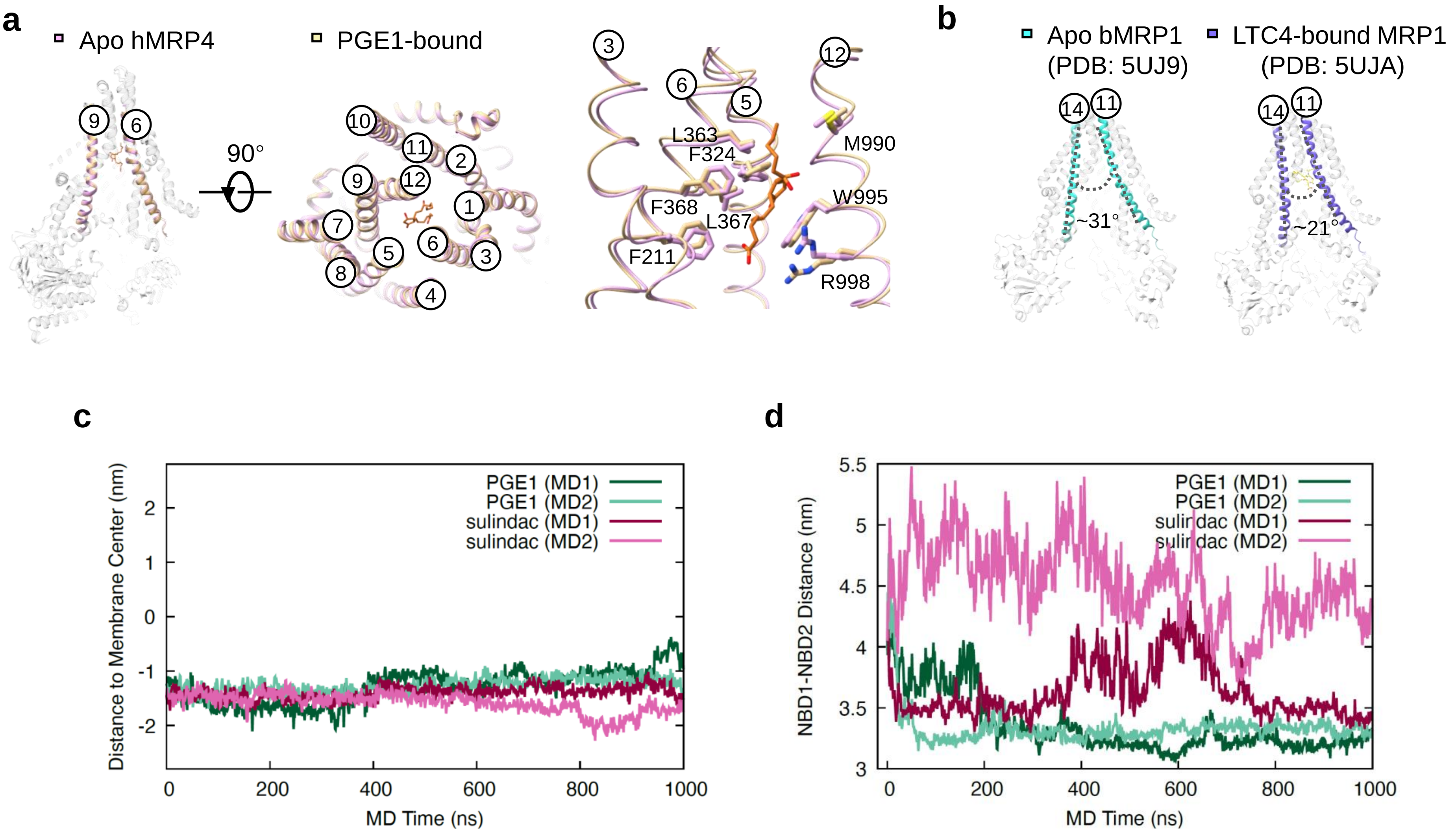

Supplementary Figure 5

Structural comparison of hMRP4 and bMRP1.

a) Comparison of the conformational changes of the transmembrane helices of the apo (pink) and PGE1-bound (burly wood) hMRP4 structures.

b) Comparison of the conformational changes of the transmembrane helices of the apo (cyan) (PDB:5UJ9) and LTC_4-bound (slate blue) (PDB:5UJA) bovine MRP1 structures.

c) Dynamics of PGE1 and sulindac in the binding pocket of hMRP4 as observed in molecular dynamics (MD) simulations.

d) Distance between NBD 1 and NBD2 in the PGE1- and sulindac-bound structures as observed in MD simulations.

Supplementary Figure 6

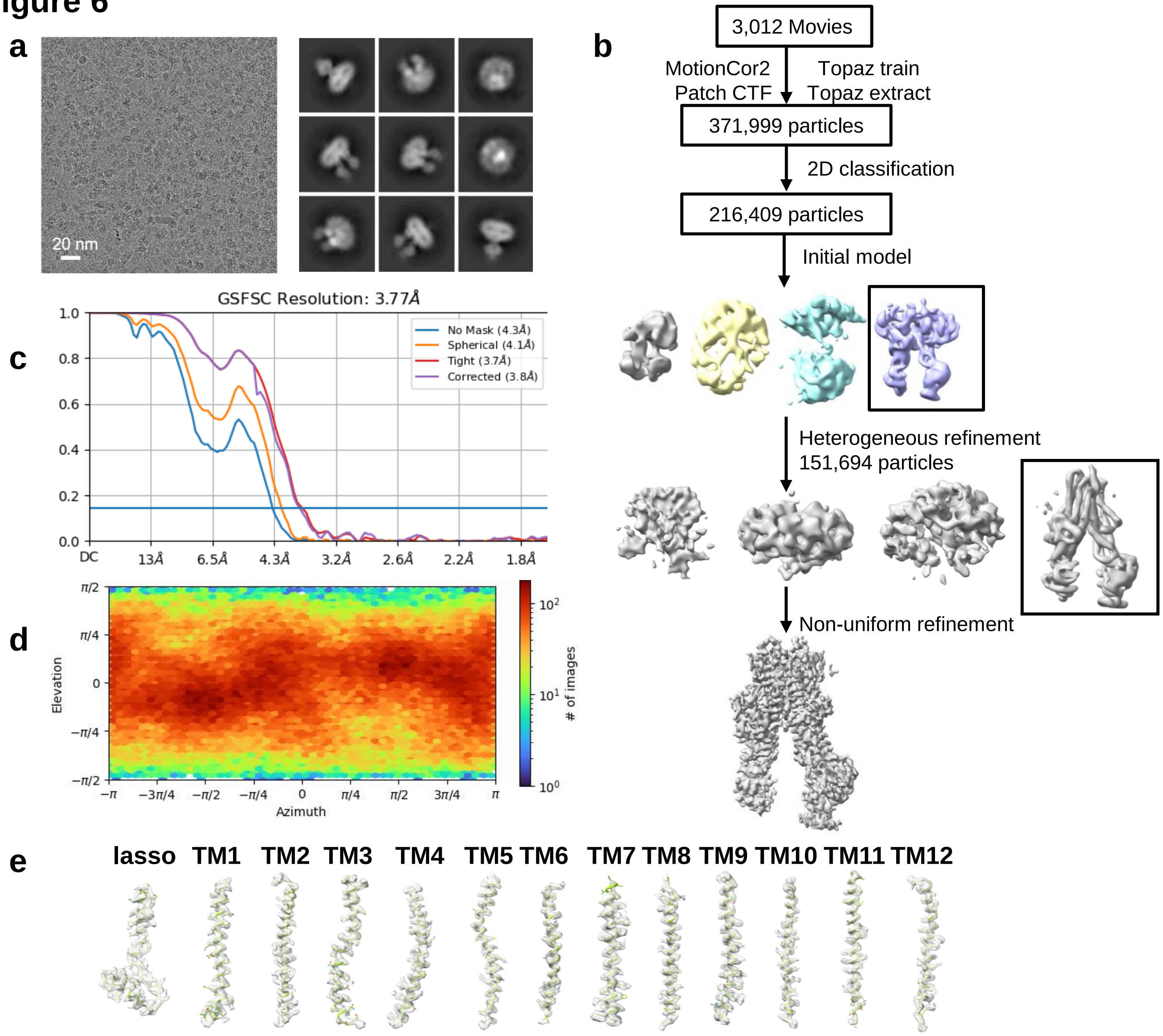

Supplementary Figure 6

Data processing and model building of the sulindac-bound hMRP4.

a) Representative cryogenic electron microscopy micrographs and two-dimensional (2D) averages. Bar: 20 nm. The micrograph is representative of 3,012 cryogenic electron microscopy images.

b) Flowchart for cryogenic electron microscopy data processing.

c) Gold-standard Fourier shell correlation (GSFSC) curve for the sulindac-bound hMRP4 map generated using cryoSPARC 3.1.

d) Euler angle distribution of the classified particles used for the final three-dimensional refinement of the overall map.

e) Electron microscopy density of lasso domain and each transmembrane helix (TM) of sulindac-bound hMRP4. Contour levels are 0.561 (lasso), 0.651 (TM1), 0.51 (TM2), 0.631 (TM3), 0.459 (TM4), 0.669 (TM5), 0.652 (TM6), 0.547 (TM7), 0.548 (TM8), 0.556 (TM9), 0.596 (TM10), 0.76 (TM11) and 0.56 (TM12).

Supplementary Figure 7 a

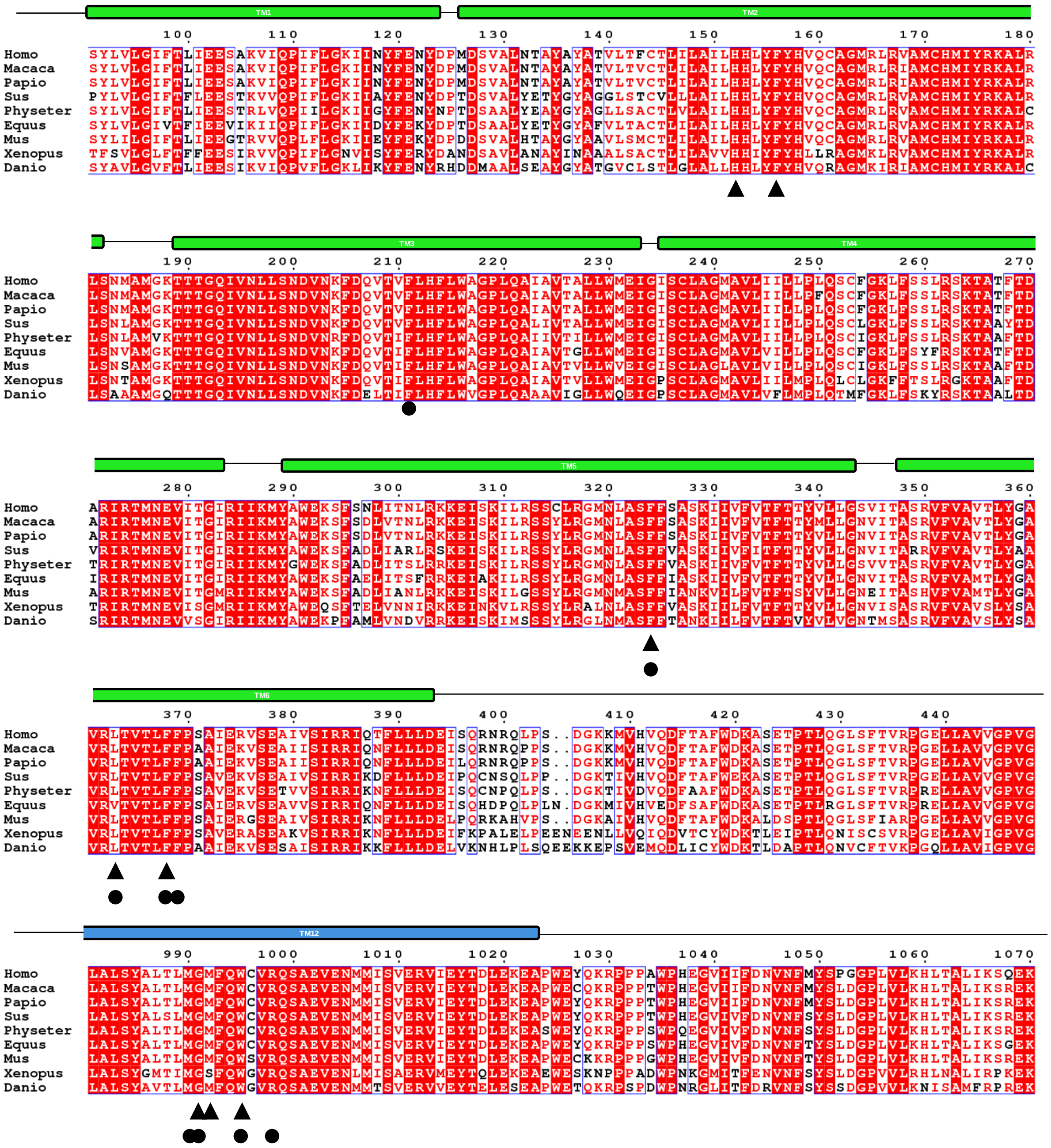

Supplementary Figure 7

b

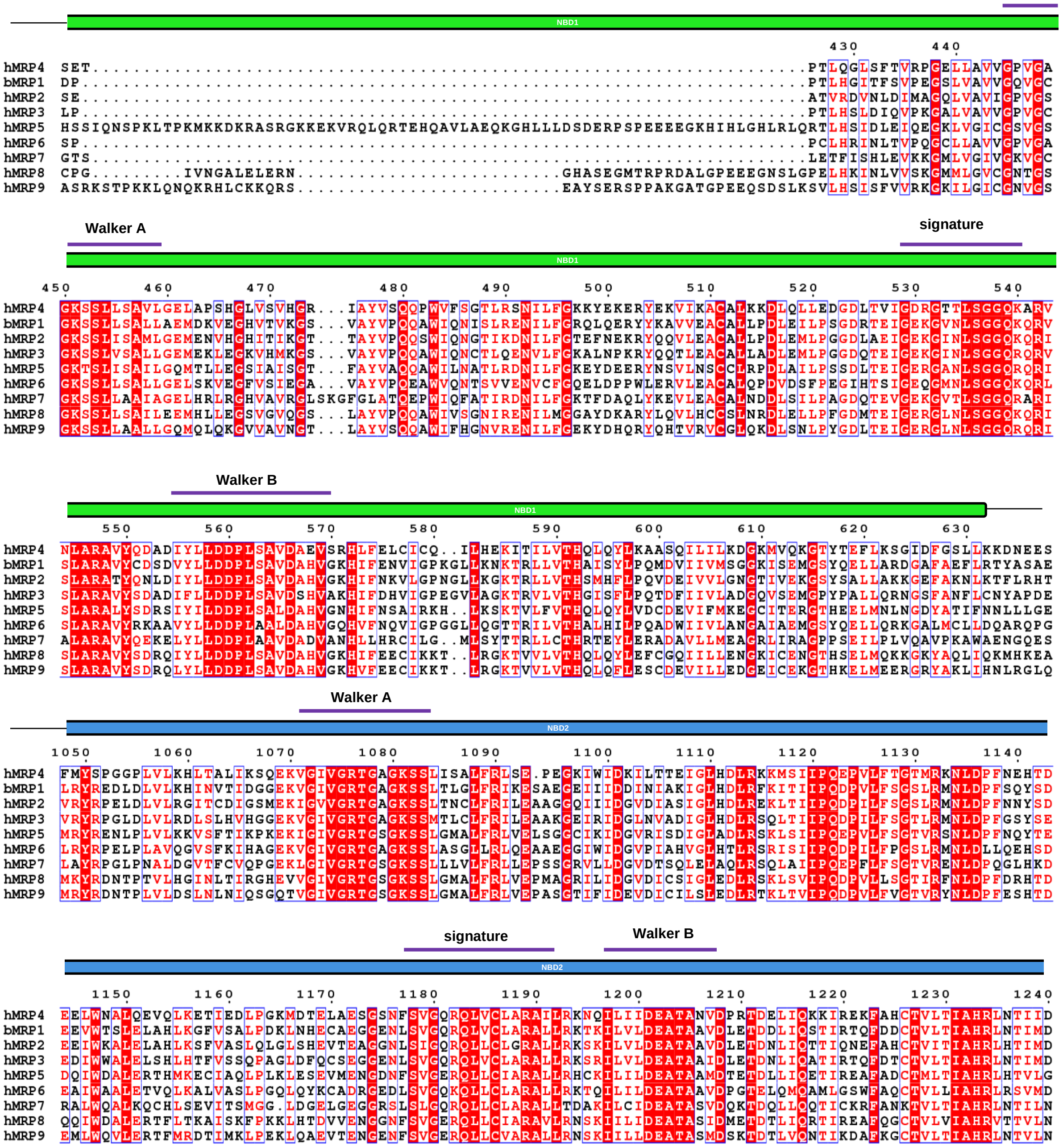

Supplementary Figure 7

MRP family sequence alignments.

a) Multiple-sequence alignment for hMRP4 and homologs. The prostaglandin E1 (PGE1)-binding sites in the substrate-bound hMRP4 structure are indicated by black circles. The sulindac-binding sites in the inhibitor-bound hMRP4 structure are indicated by black triangles. Representative secondary structural elements of hMRP4 are displayed above the sequences. Invariant and highly conserved residues are shaded red and colored red, respectively.

b) Alignment of the MRP subfamily sequences. Representative secondary structural elements of hMRP4 are displayed above the sequences. Invariant and highly conserved residues are shaded red and colored red, respectively.

Supplementary Figure 8

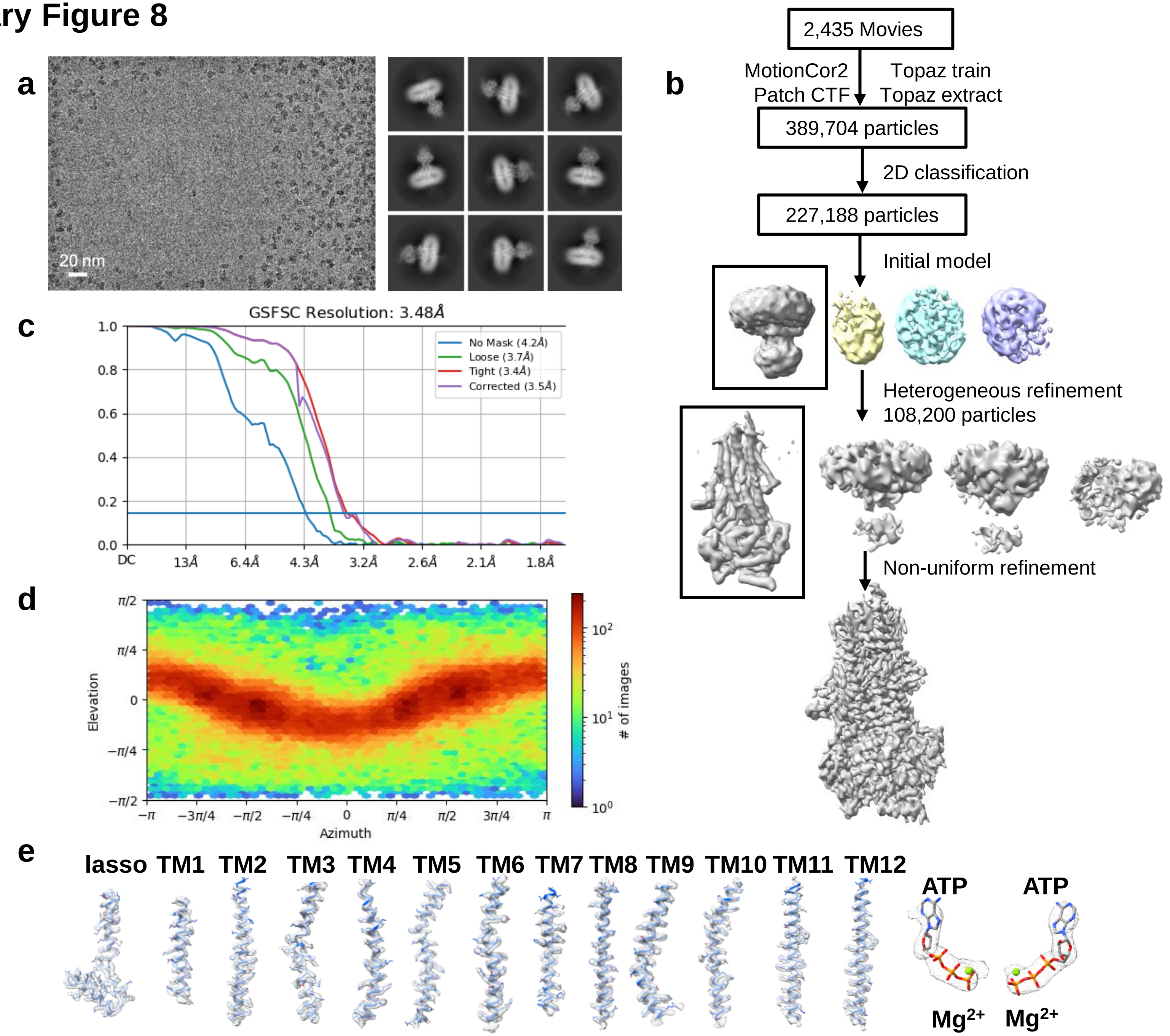

Supplementary Figure 8

Data processing and model building of the ATP-bound hMRP4.

a) Representative cryogenic electron microscopy micrographs and two-dimensional (2D) averages. Bar: 20 nm. The micrograph is a representative of 2,435 cryogenic electron microscopy images.

b) Flowchart for cryogenic electron microscopy data processing.

c) Gold-standard Fourier shell correlation (GSFSC) curve for the ATP-bound hMRP4 map generated using cryoSPARC 3.1.

d) Euler angle distribution of the classified particles used for the final three-dimensional (3D) refinement of the overall map.

e) Electron microscopy density of lasso domain and each transmembrane helix (TM) of ATP-bound hMRP4. Contour levels are 0.141 (lasso), 0.102 (TM1), 0.104 (TM2), 0.124 (TM3), 0.153 (TM4), 0.147 (TM5), 0.132 (TM6), 0.115 (TM7), 0.165 (TM8), 0.14 (TM9), 0.143 (TM10), 0.11 (TM11) and 0.113 (TM12).

Supplementary Figure 9

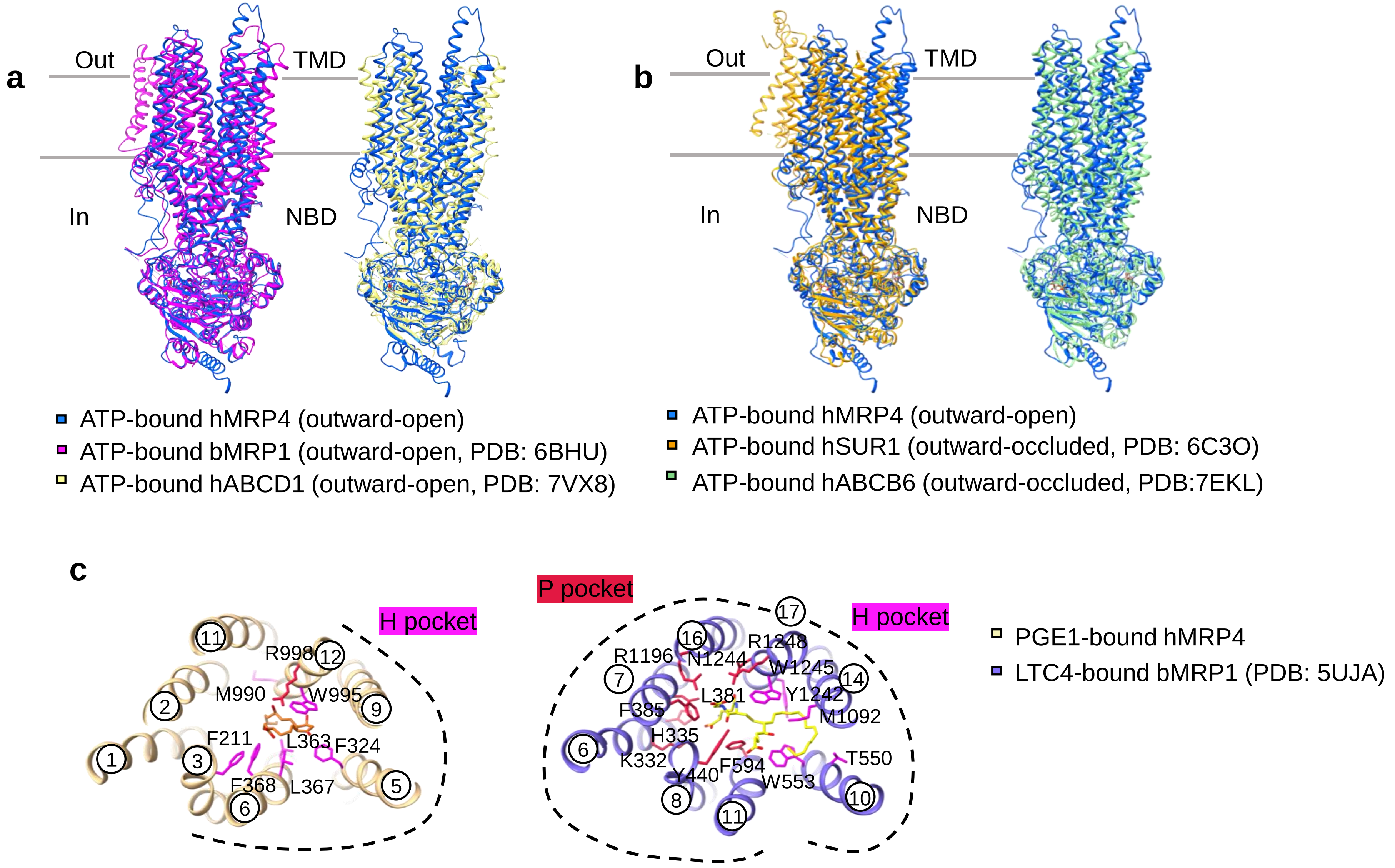

Supplementary Figure 9

Sturctural comparison among hMRP4, bMRP1 and hSUR1.

a) Alignment of ATP-bound structure of hMRP4 (blue), bMRP1 (magenta) (PDB: 6BHU) and hABCD1 (khaki) (PDB: 7VX8) and all of them show outward-open conformation.

b) Alignment of ATP-bound structure of hMRP4 and hSUR1 (orange) (PDB: 6C3O) and hABCB6 (pale green), and ATP-bound structure of hSUR1 and hABCB6 shows outward-occluded conformation.

c) Substrate binding pocket comparison between hMRP4 and bMRP1. PGE1-binding pocket of hMRP4 (burly wood) contains mainly hydrophobic pocket (left). LTC_4-binding pocket of bMRP1 (slate blue) (PDB:5UJA) contains both positively charged pocket and hydrophobic pocket (right). PGE1 and LTC_4 are colored in chocolate and yellow, respectively.

a

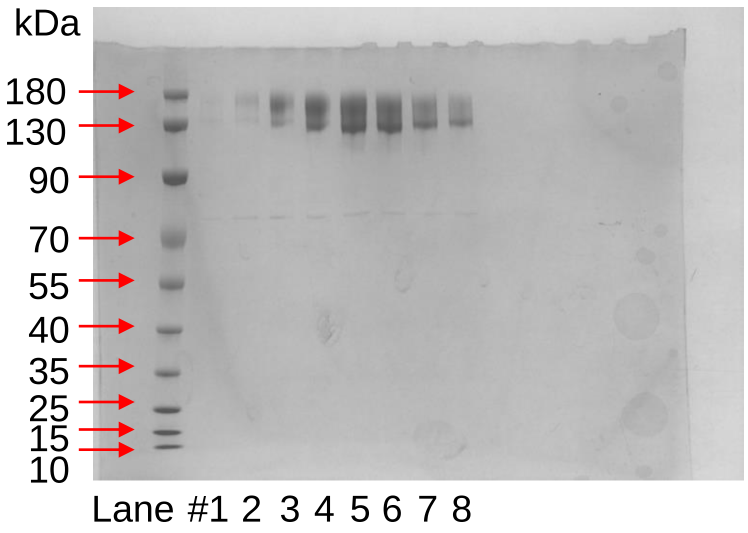

b

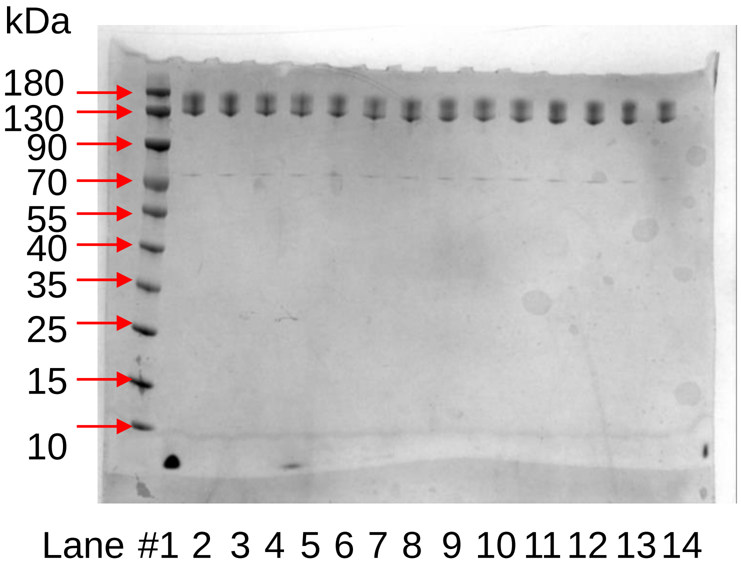

The original SDS-PAGE gel corresponds to the cropped images in Supplementary Figures 1b and c.
