# Supplementary material for: Structural basis for substrate and inhibitor recognition of human multidrug transporter MRP4
Source: Commun Biol. 2023 May 22;6:549. doi: 10.1038/s42003-023-04935-7 (PMC10202912; doi:10.1038/s42003-023-04935-7)
Supplement: Supplementary file 2 — Description of Additional Supplementary Files [file 42003_2023_4935_MOESM2_ESM.pdf]

Description of Additional Supplementary Files

File name: Supplementary Data 1

Description: List of plasmids used in the study

File name: Supplementary Data 2

Description: List of primers used in the study

File name: Supplementary Data 3

Description: Source data of ATPase activity

File name: Supplementary Movie 1

Description: The conformational change between apo and ATP-bound hMRP4.
